# The open ontology and information society

**DOI:** 10.3389/fgene.2024.1290658

**Published:** 2024-05-30

**Authors:** Meshandren Naidoo

**Affiliations:** School of Law, University of KwaZulu-Natal, Durban, South Africa

**Keywords:** information, data, governance, trust, philosophy, privacy, logic, law

## Abstract

Information, as the most elusive subject, is central to all forms of thought, governance, economic structure, science, and society. Regulation of information, especially within the healthcare field, is proving to be a difficult task globally, given the lack of a qualitative framework and understanding of the concept and properties of information (or data) itself. The presentation of the overall qualitative framework, comprising a qualitative analysis of information, data, and knowledge, will be valuable and of great assistance in delineating regulatory, ethical, and strategic trajectories. In addition, this framework provides insights (and answers) regarding (1) data privacy and protection; (2) delineations between information, data, and knowledge based on the important notion of trust; (3) a structured approach to establishing the necessary conditions for an open society and system, and the maintenance of said openness, based on the work of Karl Popper and Georg Wilhelm Friedrich Hegel; (4) an active agent approach that promotes autonomy and freedom and protects the open society; and (5) a data governance mechanism based on the work of Friedrich Hayek, which structures the current legal–ethical–financial and social society. This is insightful for questions relating to the extent of rights and duties, the extent of biological bodies and freedom, and the structure of relations in distributed networked systems. There is great value offered in this framework; furthermore, it provides critical insights and thoughts about (and uncovers the interplay between) academic culture, politics, science, society, and societal decay. Note that, in line with the ideas expressed in this manuscript, such as incorporation of personal experience (thereby mending the Kantian and Cartesian gap), a first-person perspective will be used, where relevant.

## 1 Introduction

### 1.1 Interdisciplinary fib

I will begin with a brief critical analysis of some structural axioms, which form the foundation of society and thinking today. These axioms are typically “swept-under-the-rug,” hence hiding them from criticism, while also entrenching their status, creating a closed oligopoly-like scenario. This paper aims to bring hidden links out from under the rug so that an open culture of critical reflection and change can start by reflecting upon those very foundations.

It was René Descartes (1641) who separated the (non-physical) mind (as pure thought, knowledge, objective, and universal truths) from the physical body, emotions, experiences, and empiricism. In doing so, Descartes’s body of work/thought presented two important structural axioms to the world: (1) Cartesian mind–body dualism and (2) Cartesian co-ordinates. The former is of relevance here.

In keeping with the goal of not separating thought from experience or emotions, I integrate observations and experiences of my own regarding present academic cultures. Cartesian dualism presents itself in academic culture, whereby self-experiential evidence in academic work tends to be a taboo. Another instance is that qualitative analyses, which integrate human psychology or phenomenology, are reserved mainly for philosophical, psychological, or theoretical journals. Typically, the “hard science” journals are reserved for major quantitative results that are supported by empirical evidence. Unfortunately, the idea that qualitative hypotheses or studies belong in specific locations (and not others), because the “tastes” of an audience will not be satisfied, presupposes the audience’s desires and their status as static. A preclusion of this sort serves to reduce the freedom of choice of the audience and entrenches a strong division between subject matter. This hinders the evolution of knowledge (since hidden links remain hidden in this culture). Why would literature about human psychology be any different from literature about astronomy? Does *thought* and *logic* not structure both? Previously, Copernicus believed that the Sun revolved around the Earth, thus centralizing the human as being the *universal subject* (Deutsch, 1998). Now, it seems that the human, as the observer, represents another type of universal, being the *universal object*, which is apparently removed from that of which it is a part. Both are incorrect.

Immanuel Kant then opened the doors for “relativity” (Kant demonstrated logically that the concept of the “Universal” must be limited in some way). However, Kant (1890) backtracked from the radicality within his oeuvre by positing instead that the antinomies (contradictions) of pure reason are the limits of reason, not the limits of reality itself. In this way, as accused by Hegel, Kant remained attached to pre-critical metaphysics, which posits a realm of purity, like the allegory of the Platonic cave (the truth is outside of the cave, not within it).

The Kantian Copernican revolution thus created a split between epistemology and ontology. This is a split between epistemology (as philosophy, qualitative analyses, theory, logical operations, interpretive methods, technique, structure, systems, form, objectivity, thoughts about thought itself, and universalism) and ontology (subjective knowledge, practice, content of thought, experience, phenomenology, empiricism like the sciences, and quantitative analyses). I suggest that this accurately reflects the very notion of “interdisciplinary,” which only serves to entrench (1) unfounded distinctions and (2) the Kantian axioms pervading academic culture. This is a closed system since it resists any critical analysis of its very own presuppositions/assumptions (resisting change as a result). This is contrary to what the scientific method *was supposed to be* and reflects a stifling of *imagination*, *creativity*, *and critical thought*—all of which are necessary for an open system. We often tend to think of philosophy, or to be more accurate, epistemological assumptions, as being displaced from everyday life, but we do not see how those very assumptions (from thinkers like Kant and Descartes, for example) structure everyday life, all disciplines, and all sources of knowledge.

There are two reasons for the maintenance of this fib. The first is the *raison d’etre* of capitalism, wherein relations between individuals are *treated as transactional relations between objects* (Marx, 1848; Althusser, 2014). An exemplar of the aforementioned is the logical structure of Churchillian dialectics, which involves *reciprocal causation* (Naidoo, 2023d). Churchillian dialectics is a process where subjects function as objects that freely contract with other objects in society to create/consume objects. These object relations are thought to satisfy desire and provide fulfillment (Althusser, 2014), given that these objects seemingly validate a subject’s sense of freedom of choice and the ability to contract freely. This is known as the classic liberalist interpretation of freedom—which is freedom of choice *in relation to objects*. Marx (1848) called this *commodity fetishism*.

The commodity fetish presents freedom as being tied to objects, and thus, freedom is increased where there is a greater number of objects to choose from. However, while freedom of choice *between or of objects* may be increasing, *meta-freedom*, which is the *concept of freedom of choice* itself, is *decaying.* In other words, people have more choices related to objects *but less scope to construe freedom as something else or choose among different types of freedoms.* Unfortunately, this involves a degradation of *qualitative freedom*. Increasing degradation relating to the scope of meta-freedom is axiomatic of an *unhealthy and closed society*, as Theodore Adorno (Horkheimer and Adorno, 1989) and Louis Althusser each argued in their various works. For additional information on freedom and unfreedom, choice, and the paradigm of relations between objects that characterize modern society, please refer to part E of Supplementary Material.

The second, as Sapolsky (2017) demonstrated, is that the dopamine system in the human brain does not support the capitalistic understanding of “satiation through objects.” The dopamine system uses objects for the pursuit, which is the goal (thus, this is contrary to the typical capitalistic understanding, as expressed previously). This was originally a Freudian insight, where Freud described the “objectless drives” (or the Lacanian *object-cause-of-desire*) (Naidoo, 2023b). Importantly, increases in abundance, in terms of object access, result in less uncertainty, and thus less dopamine release upon acquisition. More predictability leads to less dopamine (and hence that good feeling). Thus, neurobiology/neurochemistry does not support the notion that satisfaction is obtained through choices among material objects. Rather, satisfaction is geared toward the *pursuit of an uncertain trajectory*.

Marxist dialectic materialism is different from Churchillian dialectics, in that the former posits a *reciprocal constitution* instead of reciprocal causation (Naidoo, 2023d). This paradigm has been confirmed by scientific evidence. Sapolsky (2017) pointed out that the prefrontal cortex, as the executive region of the brain, only matures in a human’s late twenties and is more susceptible to contextual influences, as opposed to genetic ones. Furthermore, Dawkins (1976), in *The Selfish Gene*, demonstrated that phenotypes develop a kind of freedom from their genetic constituents and are more susceptible to contexts as opposed to said constituents. Dialectical materialism thus highlights the importance of *contexts* within the paradigm of *development*. Contexts, as described below, *enable more meta-degrees of freedom precisely because contexts modulate rapidly in open systems.*

In terms of the second reason for maintaining the fib, the capitalistic system requires that subjects repress, ignore, or are prevented from grasping the hidden links between different subjects, not dissimilar to the hidden variables hypothesis, or the EPR paradox, formulated by Einstein–Podolsky–Rosen (Einstein et al., 1935). For the capitalist system to maintain itself, these links *must remain hidden*. If links remain hidden, people are less likely to question the *appropriability* of the system itself.

Hegel (2018), who preceded Marx, introduced the concept of “embodied cognition” (as the substance equals the subject). This has since come to the forefront within the sciences and ethico-regulatory conversations (thanks to developments in artificial intelligence) (Juarrero, 2023). However, these discussions do not go far enough; although they demonstrate the false separation between the mind, body, and emotion; for example (Damasio, 2005), they do not seek to mend the Kantian split. Lastly, the maintenance of this fib, and the entrenchment of a strict divide of the disciplines, serves to hamper the cohesion of the overall total distributed network, which is that of knowledge acquisition (described below). To assist the reader, a brief navigational map is included in part G of the Supplementary Material.

## 2 Knowledge society

### 2.1 Enlightenment and the knowledge enterprise

The European Enlightenment greatly impacted the foundations of all system-building, including legal, economic, social, political, financial, ethical, religious, and scientific systems. The European Enlightenment took three main forms: (1) a political thesis for better governance; (2) a philosophical thesis for a secular foundation based on rationality and science; and (3) an economic thesis on creating more wealth (Mokyr, 2012).

What allows knowledge, ideas, and thought to flourish is an open society (Popper, 1945; Thaldar, 2017). An open society is one in which the thoughts of mad men, who go against the grain, are not rejected or punished as they were in 17th- and 18th-century Britain. These rebels must be protected and afforded the space to be the mad men that they are to protect the openness of a society; it is the acts of such mad men that *ensure that society reflects upon itself.* As described by Žižek (2003) and Naidoo (2023a), it was Immanuel Kant who put the first crack in the concept of the Universal, the infinite, or the concept of “objective,” followed by others, such as Hegel (2010) (a complete ontology *of incompleteness*), Karl Marx (1848) (false consciousness), and in mathematics, Gödel (a conditional mathematics), George Cantor (the Cantor set describes a limited infinity), and Hans Peter Luhn (the computer science concept of hashing, which is analogous to the Cantor set) (Naidoo, 2023b).

The core of *liberalism* stemmed from the above, which is the *marketplace of ideas* (the marketplace is the necessary condition for the social contract). In the marketplace, ideas compete, fight, coalesce, triumph, and hibernate. The marketplace has a structure that reflects varying interests, like economic, social, and political interests (Mokyr, 2012). None of these interests solely determine outcomes within the marketplace, and each interest typically has varying degrees of importance; there is thus another marketplace within the marketplace. The ultimate marketplace, within which all others are nested and structured, is the *theory of reality*. The ultimate boundaries of a marketplace are thus ideological and based on theories of knowledge and modes of structuring or validating said knowledge. Each mode or theory turns on questions like, what makes knowledge possible? What is persuasive? What kinds of evidence and logic are possible? What kinds of experiments are necessary? How do we structure what the content of truth is? What does it mean to be correct? What is a true statement?

To provide answers or to construct new questions and answers, it is important to determine whether the structures of any given marketplace being visited are suitable or unsuitable. Typically, debates on this issue lead to reflections involving the Industrial Revolution and the Enlightenment (Mokyr, 2012). The English Enlightenment concerned the removal of ancient and conservative governance structures and values. However, this new modern form seems to have just translated some of the older values into a different language (Mokyr, 2012). The English Enlightenment was not in opposition to Protestantism; rather, it took the Protestant ethics and created a new, secular society around it. The new capitalist society is Protestant, often without knowing it. Herein, a liberal form of Christianity was used to justify the pursuit of one’s self-interests, which perfectly suited the industrial epoch. Hence, science, politics, law, ethics, religion, and the like are shaped by the same schema.

### 2.2 Scientific method

In the 18th century, leading up to the invention of the steam engine, science was very different from today. The common quip is that the invention of the steam engine did more for science than science did for the invention of the steam engine. This “pre-modern” science shaped important developments within empiricism, which became known as the “scientific method.”

There were many important discoveries in the sciences, such as formulae and understandings relating to heat, thermodynamics, and electricity (Rosen, 2010), which catalyzed perspective shifts as to how the world, physics, and chemistry worked. Given these discoveries and the “force model” imposed by Newton, causation and determinism were the dominant ideas of reality. This catalyzed a move away from the teleological purposiveness, inherent to Aristotelian philosophy and methodology (Naidoo, 2023d).

The scientific endeavor involved testing conclusions, ideas, and premises and falsifying non-repeated results since science, at this time, believed that invariance and physics should dominate knowledge validation and status as such (given the huge successes of the cause–effect relations of Newton’s force models). What science sought out was evidence in the search for knowledge, and in doing so, it also moved away from the influence and dominance of the Catholic Church. From this cultural shift, the concept of “replication” was born, and inventing was viewed as a social enterprise, instead of a private one. Previously, inventing was seen as the practice of a “lone genius” (Rosen, 2010). The currency for inventors during this time was mostly recognition or fame (Rosen, 2010).

The notion of perfection, ideal, objective, or universal, at least in Western scholarship, traces back to Plato (2002) and his World of Ideas and Pure Forms. Kant and others built off these ideas through the introduction of *a priori* pure forms which structure the mind, experience, and thought.

Technological advancements in this period were nominal, mostly consisting of simple improvements on instrumentation and navigational tools (Rosen, 2010). However, there were many novel understandings, scientific principles, formulae, and logical deductions that were understood as *a priori* knowledge. Society at that time, did not benefit from the unearthing of *a priori* concepts. In other words, no tangible value in the form of usable forms of technology was acquired from their derivation. Developing tangible or usable forms required a lot of time and other resources, much more than one individual could possibly provide. Hence, inventing became a social endeavor, wherein many could collaborate, thus sharing resources and responsibilities in the production process (enhancing usefulness, accessing, recording, storing, working, and exploring different avenues). This is what a distributed, networked system is. Hence, the scientific method sought *value* in the *form of physical objects*, and the production of the said value required bridges to be built between *a priori* concepts and utility; theory and empiricism; and theorists and artisans (Rosen, 2010; Mokyr, 2012). The scientific method, as the vital cog, transformed the Age of Enlightenment into the age of utility, objects, and the later Industrial Revolution. The purpose of the formation of the 1836 Select Committee on Arts and Manufactures (Sproll, 1994) was for the committee to determine the best ways in which to disseminate knowledge and principles in the fields of knowledge production and that of makers or manufacturers (Sherman and Bently, 2003). The singular value of *utility through the transformation of knowledge into physical objects/products* was siphoned into the jurisprudence of patent law. Inventing, in law, was now solidified as a social enterprise, which included the *acquisition and validation of data and knowledge*. The dominant paradigm prior to this Enlightenment was that of the Lockean private property. In this view, knowledge was understood to be within the personal domain of a person and thus private. Importantly, this underlies the typical conception of personal identity and privacy, which many are unaware of being traced back to Locke. The purpose of the Enlightenment was to bring out, and ensure transparency and access of/to information and knowledge, which was hidden away (hence the name “Enlightenment”) under the veils of preceding religious and cultural practices (Dolar, 1991). This was the first move toward an open society, based on information and knowledge being freely accessible.

The scientific method ushered in a new understanding: to enhance conversion and utility, knowledge and data had to be recorded and shared for testing. Knowledge was then understood to be conditional on trust and not absolute (Rosen, 2010). Knowledge and conclusions could be improved and replaced. No longer did logic hold center stage as the singular source of validation. Experimentation became the defining methodology in the social enterprise of knowledge (Rosen, 2010).

Unfortunately, the “practical” or utility justification of innovation pervades many academic explorations, and the necessity and difficulties associated with obtaining funding only serve to entrench four fibs: (1) scientific empirical *experiments* are necessary to validate knowledge; (2) there is a strong separation between “thinking” and “doing.” As demonstrated by thinkers such as Kant, Hegel, Freud, and Marx, knowledge can be validated through thought alone (second-order inferences). Sapolsky (2017) also echoes the argument that one does not need to perform empirical experiments to simply confirm something that is observationally clear. Fib (3) is that there is a strong separation or difference between science and religion. The final fib (4) is that there is such a thing as *progress*. Taleb (2007) demonstrates that progress is not a *linear* concept (nor averageable, much like knowledge), nor is there any consensus as to what constitutes progress. Given that neither progress nor knowledge is linear, both are impossible to evaluate in extremist systems.

## 3 Freedom and surprise

### 3.1 Semantics and meaning

The breakthrough of communications theory, later dubbed “information theory,” occurred when Claude Shannon and Weaver (1964) published their book *The Mathematical Theory of Communication*. Both felt it was a mathematical theory of communication, as opposed to a theory of information. The mathematical formulations therein describe the necessary conditions for communication using concepts like symbols, signals, and carriers. Therein, Weaver described information as the measure of one’s degree of *freedom of choice* when selecting a message (Shannon and Weaver, 1964).

Shannon and Weaver (1964) were concerned with *transmitting information,* and not *meaning*. Thus, Shannon created a formula that could transmit messages in the presence of *noise*. This formula for encoding messages with maximum efficacy was the same as the one created by Ludwig Boltzmann half a century earlier (Hidalgo, 2015). Both formulas treated *information as physical*. For Shannon, informational entropy is the *minimum volume of data necessary to specify any type of message* (Shannon and Weaver, 1964).

In this paradigm, information is *meaningless* (and meaning is not information); *it is the receiver/interpreter/perceiver who weaves-in/transmits meaning* into information. It is *not valuable* either. Meaning and information are often confused because of this automatic (unconscious) transposition (Taleb, 2007). This creates the illusion of a hidden depth within the information. This automatic transposition occurs because of a need *to reduce information and conserve energy*. Thus, meaning is not the message, and meaning lies within the receiver, contexts, and prior knowledge (Hidalgo, 2015). Meaning is a tool used to communicate the physical order of things.

Meaning (dubbed semantics, from here, as semantics are associations of meaning) exists in various spaces and times, within everyday life, and history. Depending on the spatial, temporal, or spatiotemporal co-ordinates, the semantic content of each co-ordinate will vary. Different co-ordinates or contexts entail different semantics, and *vice versa*. Hence, semantics and co-ordinates are co-constitutive (Naidoo, 2023d); semantics constitute spatiotemporal coordinates as much as spatiotemporal constitutes semantics. Semantics are also not just the product of, or constituted by, human brains, but rather exist as varying types of constraints, which can be context-dependent or context-independent (Naidoo, 2023d). Nonetheless, human beings do bias semantics within spatiotemporal co-ordinates, within their favor (as individuals or as a species), to maintain their viability and structure relations within a networked, distributed system. The semantic content of a co-ordinate is constructed through processes of trial and error and jury rigging, testing, validation, correction, updating, and rejection. These processes require assessment criteria, which bias some semantic content over others, known as *selection criteria.* Selection criteria are also the product of context-dependent or context-independent constraints. For example, human society would not designate that a volcano is a semantically suitable location for a school, but in human history, it was a suitable location (the heat and noxious fumes are context-independent constraints) for sacrificing virgins to appease the gods. Hence, the semantics of contexts shape human selections.

What is required to maintain the semantic content of a spatiotemporal location is continuous, repeated observations and assessments, known as *measurements*. It was Sigmund Freud (Naidoo, 2023b) who first suggested that repetition is not something that is defined by humans but something that defines humans (Lacan, 1979). Hence, the *validity* or *continuous appropriability* of semantic content is determined through repeated observational or experiential assessment (measurements) consisting of taking in, keeping up-to-date, and updating factual information in the form of *events*. Importantly, events are not removed from observation; observation and events are reciprocally constitutive.

### 3.2 Passive and active mind

Understanding of David Chalmers’ (1996; 2010) “hard problem of consciousness” requires knowledge of what preceded the hard problem. In 1799, Friedrich Heinrich Jacobi (di Giovanni and Livieri, 2018) wrote a letter to Johann Gottlieb Fichte in which he expressed his unhappiness about the loss of subjectivity, which arose because of Spinozism and the rationalistic physical sciences. Jacobi wanted to “save” humanity from the perils of nihilism, tasking Fichte, as the “true disciple” of Kant, with this duty (di Giovanni and Livieri, 2018). In *The Spinoza Letters* (Spinoza, 1995), Jacobi quite clearly foresaw that humanity would be saved by a return to inner experience and feeling (affect) (di Giovanni and Livieri, 2018). However, thoughts like Fichte’s only served to entrench nihilism, which Hegel argued, since there was a reliance on “spurious infinities,” such as the linear flow of time or advancement. Neither time nor progression is linear (Deutsch, 1998).

An important development of the human mind (and of identity) was John Locke’s (1860) *An Essay Concerning Human Understanding*, wherein Locke proposed an account of the passive mind as a tabula rasa. Locke’s mind simply served to reflect what was perceived. This passive mind was challenged by others, like Freud, who proposed, in his topological economic theory of mind, that the mind was active. In Freud’s (1915) breakthrough work, *The Interpretation of Dreams*, he proposed that the separation of the conscious and the unconscious was a *defensive threshold*, implemented by the psyche, to protect against high energy levels of states and excitation. The purpose of this defense was to *maintain a dynamic equilibrium/homeostatic stability*. The mind *is active*. Consciousness and unconsciousness were separated because excessive energy is damaging; the unconscious, which contains the highly energy-invested (*bestsum*) forms of thought and ideas, *is limited by consciousness*. In other words, *consciousness exists as nothing but a limit to energy.* These views have been confirmed by modern science (refer to part D of Supplementary Material).

**FIGURE 1:**
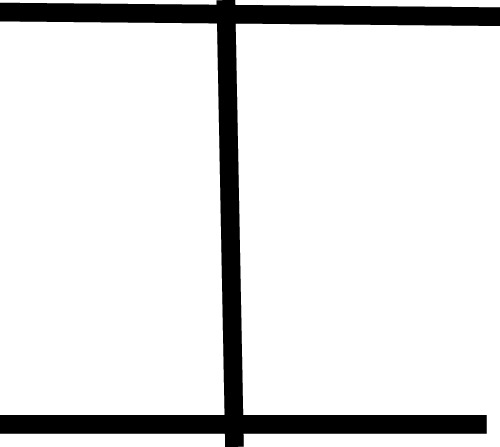
Composed by author, 2023.

Chalmers (1996; 2010) also queried why there exists the subjective “I.” Naidoo (2023a; 2023c), relying on Lacanian thought, and Benveniste (1966) noted that the “I” *is a stabilizing referent*. The purpose of the “I” is to indicate the spatial location/identity of the speaker. At a deeper level, and importantly, the subjective “I” represents *a resistance to symbolic representation*, meaning a failure to obtain a positive identity through positive knowledge of *what the* “*I*” *is.* The resistance to symbolic incorporation (a deadlock or knot) is important for maintaining an open system and society, as argued by Althusser (2014) in his critique of the overdetermining effect of state apparatus. In other words, the “I” serves as an irreducible, indivisible link enabling constitutive couplings, interrelations, and entanglements that serve to create an autocatalytic feedback loop between a system and its previous context [which now becomes a “niche” (Naidoo, 2023d)]. This constitutive coupling enables for the structuring of language, identity, and knowledge. Indivisible knots are used to construct distributed, networked systems and maintain their status as “open,” which is a dynamical (non)equilibrium state of stability (homeostasis). The content of the “I” is “unknowledge,” meaning that there is no positive knowledge as to *what it is*, but rather knowledge *as to what it is not.* Consensus on the “I” is thus reached through *determinate negation*, instead of *affirmation*. Consensus is thus constituted through *a lack of positive affirmation.* I call this constitutive consensus dis-consensus, which in social matters takes the form of “we agree to disagree.” Importantly, this kind of reasoning is analogous to the Kantian *infinite judgment*, the Gödel incompleteness theorem, or the Hegelian logic of *Aufhebung* (or chirality, present in biology and chemistry) (Naidoo, 2023b). To examine the appearance of the “I,” we see that it is constituted by two parallel horizontal lines and a single perpendicular line joining the two, as in Figure 1 below.

**FIGURE 2:**
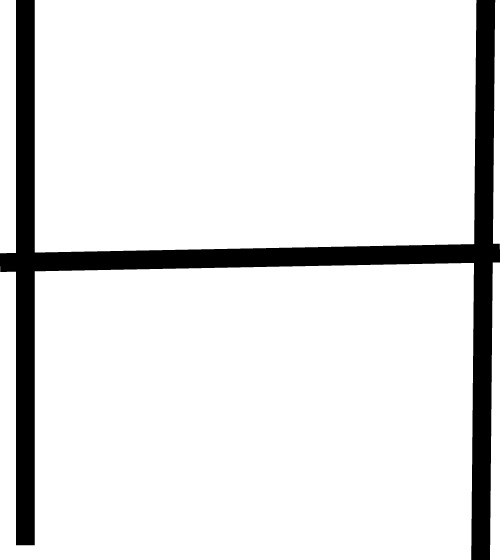
Composed by author, 2023.

In other words, the parallel lines are kept apart but also kept together by a vertical line, which separates and joins them. Returning to Hegel (2010), this is precisely the logic of *Aufhebung,* or chirality, which describes the holding apart, while simultaneously holding together something. They are held together by what I (based on Lacan) call a pure difference (Naidoo, 2023b). That pure difference is an indivisible link, as described. When turned on its side, it resembles Figure 2, transitioning from the subjective “I” (also the “I” of information) to the “H” of homeostasis.

**FIGURE 3:**
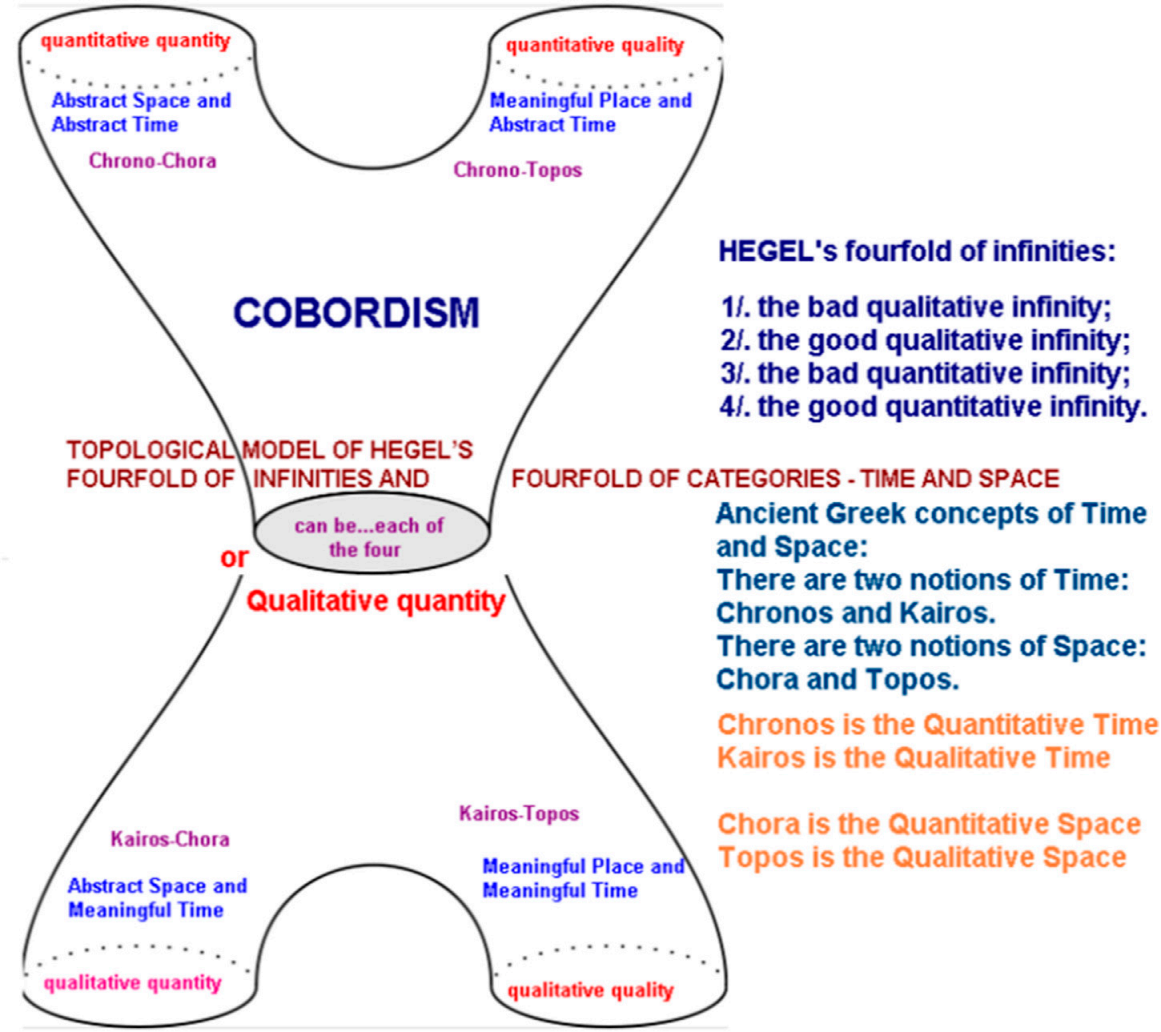
Dimitrov (2015).

**FIGURE 4:**
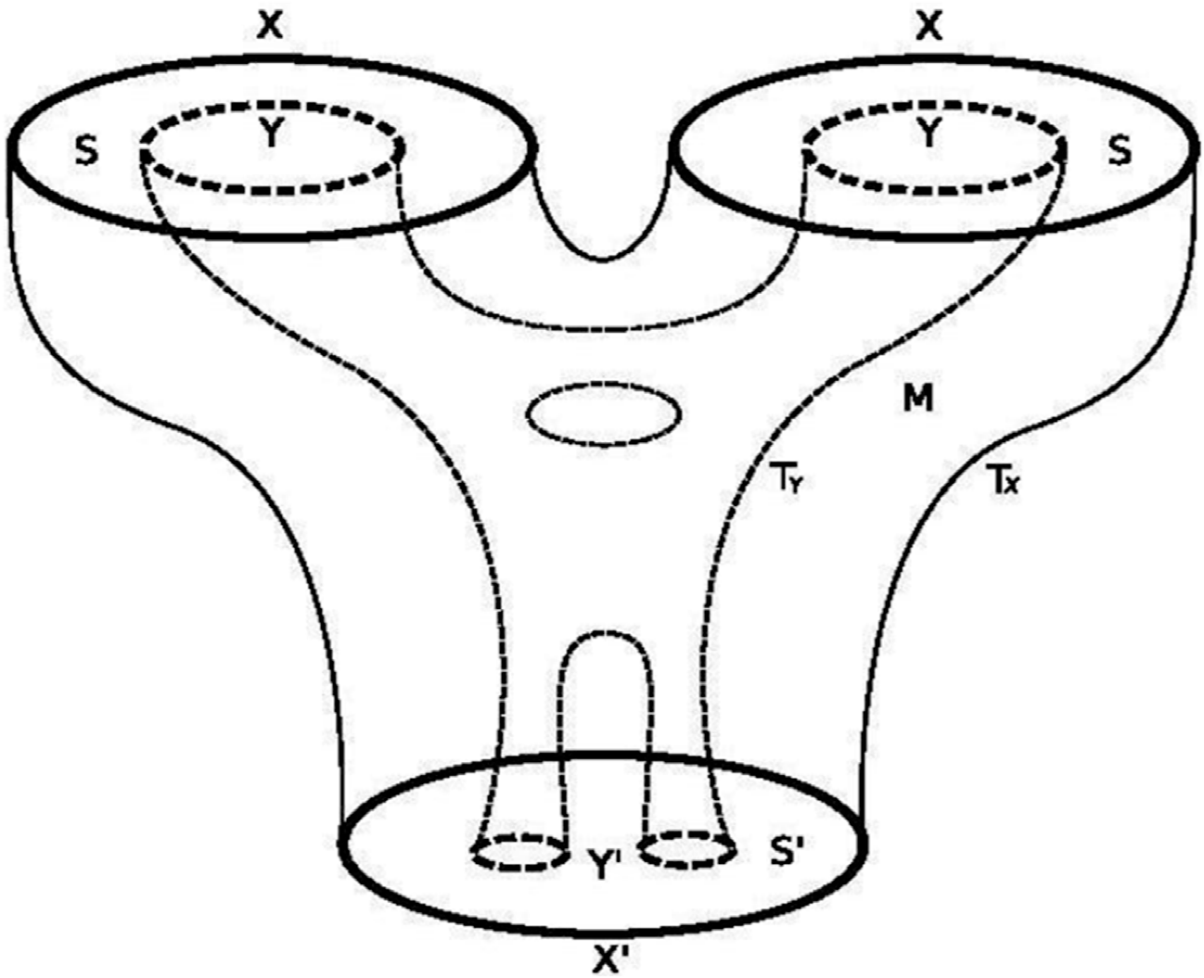
Dimitrov (2015).

The logic of *Aufhebung* (Naidoo, 2023b) is coupled with that of an autocatalytic-feedback-loop, akin to the Belousov–Zhabotinsky chemical reaction, wherein the presence of a fourth step (the observer in the sciences) encloses an open-dynamism intrinsic to a system (Juarrero, 2023). Such a logic provides the necessary enabling conditions for a system to incorporate contextual information into its very constitution, increasing its informational content (Yoshida, 2010). The Hegelian dialectic is topological (relating to circles)—being similar mathematically to Poincare’s cobordism, which he explained in *Papers on Topology: Analysis Situs and Its Five Supplements* (Poincaré, 2010). The four categories of cobordism are almost mirrored by Hegel’s four-fold infinities (Naidoo, 2023b). Cobordism describes how two circles can be morphed into two other circles, like a pair of pants (Dimitrov, 2015). In Figure 3, the Hegelian fourfold infinities are represented. In Figure 4, the Hegelian ontology, which produces an “internal pair of pants” within the original, is presented. Figure 3 represents what is known as *autopoiesis*, *self-causing logic*, *or Aufhebung*.

Both *Aufhebung* and autocatalytic feedback loops are necessary conditions for open systems, open societies, and the creation of a dynamic equilibrium (a stable state of non-equilibrium, or homeostasis). It is necessary to note that knowledge is not static, but dynamic and evolving, in such systems. On a neurobiological level, the subjective state, as pointed out by Sapolsky (2017) and Naidoo (2023b), operates as a simulated, stochastic risk management strategy aimed at maintaining the viability of the system itself.

### 3.3 Information gain and surprise

Information is a measure of the *degree of surprise* obtained by an agent who observes or experiences an event (thus the link to probability). This degree of surprise of an event is described by *self-information*. Information (I) is *thus a function* called self-information. Self-information is the informational content inherent to any event or occurrence. *Information entropy* extends this idea to discrete random variables (X). The entropy of (X) is the *average of self-information over all possible outcomes of (X).* Conversely, the entropy of a random variable describes *the average degree of surprise obtained by the outcome* of (X). There is an increased information gain after a surprise, as opposed to expected or predicted events. Information gain is tied to *reductions in entropy*. Entropy, to conclude, is about the degree of surprise obtained from outcomes based on prediction (part B of Supplementary Material).

## 4 Building an ontology

### 4.1 Negative definition

Any ontology needs to begin with a definition; so, *what is information?* Currently, there is no *qualitative* consensus on an account of information; only a compendium of various and vague axioms exists (Floridi, 2009). We know that information is quantifiable, additive, storable, and transmittable. It is also a golden thread that runs through all disciplines (Deacon, 2007). It is certainly incorporeal and intangible because one cannot physically handle or manipulate information. In the 21st century, the importance of information increased, resulting in heavy commodification (Badiou, 2006; Hidalgo, 2015). Information was thought to be physical because of its *physical embodiment*. It was not construed as a “thing,” but instead *a physical arrangement*, taking the form of a thing. Information was that which differed from its surroundings, based on its *identity*, which is its *appearance*, or the *physical order of its arrangement* (Hidalgo, 2015). As time passed, the nature of information morphed into being understood as digital, immaterial, and non-physical.

A founding father of cybernetics, Norbert Wiener (1961), provided a *negative* (exclusionary) definition of information in *cybernetics*. Wiener (1961) said that

“…The mechanical brain does not secrete thought “as the liver does bile,” as the earlier materialists claimed, nor does it put it out in the form of energy, as the muscle puts out its activity. Information is information, not matter or energy. No materialism which does not admit this can survive at the present day.”

The last part of this quote is insightful; *information is not matter or energy*. This is a negative definition since Wiener does not propose to know what information is, but he suggests *what it is not*. Although information is neither matter nor energy, *it needs matter for embodiment and energy for its communication*.

### 4.2 Reduction, patterns, and data

Entropy is a limit on efficient communication of the outcome of (X). In other words, it describes how much compression can take place while maintaining the efficiency of the communication. In communication theory, there is a correspondence between the base of the logarithm and the symbol quantity, which is used in a hypothetical scenario involving two agents who communicate surprising, random events (Brownlee, 2019). Any change in the base of the logarithm results in a corresponding change in the entropy equation (Bernstein, 2020). This means that informational entropy describes the minimum number of symbols (lower bound) needed to communicate an outcome of (X). The base of the logarithm of the self-information function is also the lower bound on the number of symbols required to communicate, as above.

Information is expensive (energy-wise) (Taleb, 2007) to obtain (the rule-finding, updating, learning, and executive region of the human brain, being the prefrontal cortex, is highly metabolic) (Sapolsky, 2017). It is similarly expensive to store, order, manipulate, and retrieve information. To combat this, systems need energy-efficient ways to handle and store information. The solution to the energy-efficiency issue is to order information, thus making information less random, which requires an association or attachment to narrations, words, or symbols (Taleb, 2007). As Shannon and Weaver (1964) demonstrated, for efficient communication, strings of symbols and signals can be used. Shannon (1940) also demonstrated that some symbols will be used *more frequently* than others (the source of the code will have a higher frequency with regard to certain symbols or knowledge). Based on this, it was possible to assign shorter codes to more frequently occurring symbols (including symbol pairings), reducing the total length of the required code. Natural language processing (in artificial intelligence) uses the same principle, namely, the frequency of a letter depends on what precedes the said letter. This is Shannon’s empirical entropic frequency distribution formula (Hartnett, 2022). This is *a predictive functionality*. Importantly, Hawkins and Dawkins (2021) demonstrated that the brain functions according to a single cortical algorithm—*which is prediction*. Humans build their own subjective (and inter-subjective) world-maps according to this system, comprising spatiotemporal semantic relations.

In other words, *compression* is fundamental for energy efficiency. Compression is achieved through the creation of tunnels, called narratives, wherein the *dimensionality* of information is reduced (Taleb, 2007). A side effect of compression or reduction is that vast chunks of information are typically ignored. Along with reduction, compression requires an active function of repeated, continuous assessment and observation, wherein information is abstracted (reduced in dimensionality). The abstracted information is reduced through the combination of both ignoring chunks and the creation of narrations (association links or chains of sequential chunks of observed events). These selective narrations, as sequential observations, are known as *patterns*, which connect events, thus *making prediction possible*.

Patterns are abstractions or *representations of reduced information*. Abstraction (as opposed to concretization) is fundamental to mathematics and physics. Abstraction describes the derivation of non-physical patterns. Concretization refers to the creation of physical objects. Abstraction is similar to Plato’s (2002) concept of *ideal*. The transformation of the Platonic ideal into the language of sciences by philosophy is important to understand. It is this concept that structures others, like Newton’s universal clock. The Platonic and Aristotelian triadic structure consists of three components: (1) the physical/material world; (2) the mental world; and (3) the world of structures. Abstraction is thus the *mental process* of removing properties from an (X), followed by attaching a name/identity to those properties. These abstractions belong in the *world of mentality*, which can be *individualized or collectivized*.

Patterns enable efficient informational manipulation and storage. It is impossible to use and store all available information, given the several orders of magnitude of energy required. Hence, only useful bits or patterns are used to make generalized knowledge possible. Examples/descriptions of patterns include summaries, compressions, narrations, episodes, sequences, slices, and foliations of information. From patterns, rules or laws can be derived, which are generalized forms of knowledge (Woodward, 2000). Generalized knowledge is that which is judged to be invariant (Woodward, 2000) for a given set of contexts, thus holding the status of governing or ruling constraints (Juarero, 2023), which allows for greater predictability, less uncertainty, and the performance of experiments (which test and maintain the validity of the said rule or law).

Rules and laws, as governing constraints, are compact, and because of their reliability value (Woodward, 2000), they enable functions to occur with less energy expenditure, while also enabling coherence, comprehensibility, and understanding. Media, in the forms of books, magazines, plays, stories, videos, movies, paintings, poetry, and all sciences are based on this principle of compressed bits of information (Taleb, 2007). Patterns also take the form of *ideas or concepts*.

The name of this compressed information, such as patterns, ideas, concepts, episodes, movies, or any of the aforementioned, is *data*. Data describe *identifiable*, embodied, encoded, or nested patterns within various physical mediums, structures, or forms (like vehicles). In a non-physical medium, such as a mind, patterns or data exist in the form of ideas, concepts, or thoughts, each of which is largely represented by analog encoding through synchronized neuronal spiking and synaptic connections in the brain (Hawkins and Dawkins, 2021). These sequences form through continuous, repetitive observations. As Hawkins and Dawkins (2021) note, world maps are built up of reference frames (mental structures), and thinking is a virtual movement through reference frames.

Observation, I suggest, *is a dual function*. The *passive form* of observation is known as measurement, quantizing, foliation, and hermeneutics. These are repeated processes of *automatic assessments and updates*. These passive observations consist of subjective, yet autonomic behaviors, aimed at updating and maintaining a system’s world map (Hawkins and Dawkins, 2021). In doing so, these processes maintain the system’s dynamic equilibrium. Observations are thus subjectively sliced into qualitative and quantitative “pieces” of space, time, or a combination of both. Passive observation can occur through perception and experience (if it is possible to even distinguish between them). Observation is thus precisely a method of *biasing information in favor of the observer*.

Embodied knowledge, as passive observation, is difficult to acquire, communicate, store, and copy. The knowledge and knowhow contained in the human body (and mind) is “heavier”; knowledge and knowhow contained in objects is *relativity easier to move*, as objects can be carried and communicated through mediums, such as books and the internet (Hidalgo, 2015). Embodied knowledge is comparatively slower to acquire. This includes technical proficiency like scientific, programming, and legal skills/techniques. It is known as *expertise*. It includes the knowledge and abilities of other team members and knowledge of contextual circumstances. Embodied knowledge is *biased in terms of sociality*; it is accumulated and translated through *social learning and experience*. Beginners learn from more experienced persons; thus, it is not an individual endeavor. There is a social and experiential learning curve, which makes its accumulation time-consuming and *limits* the speed at which individuals can develop it. Embodied knowledge also biases geographic locations, which have greater quantities of some quality. To solve the issue of embodied knowledge distribution, society breaks up knowledge and knowhow among different individuals in a distributed, networked structure.

These individuals then utilize their specific forms of knowledge and knowhow *as a social network of individuals performing as a team* (Hidalgo, 2015). Through networks, the collective body of this knowledge and knowhow *can be increased*, which is *greater than what an individual can produce*. Importantly, these networks must be able to distribute knowledge evenly and ensure there is cohesion and a combination of individual parts to produce the result. It is harder to maintain a cohesion rather than to ensure everyone performs their roles. *The whole is thus greater than the sum of its parts*. For this, there must be timeous and *performative cohesion*, *shared responsibility*, *social practice*, *updates*, *corrections of mistakes*, *assessments, proper communication*, *and trust*.

The Freudian “id” described the *automatic*, unconscious aspect of Freud’s topological mind. In modern terms, this would describe the *lizard brain*, which performs *autonomic* functions like maintaining homeostasis in the body (Sapolsky, 2017). As I have described, patterns or *id*eas describe subjective (or relative) perspectives intrinsic to the constitution of any pattern or data. In other words, *id*eas are inherently linked to the concept of *id*entity. The processes of observation and abstraction described above are automatic functions of the brain and body (Hawkins and Dawkins, 2021). Both observation and abstraction aim to maintain autonomic stability or homeostasis (Sapolsky, 2017). These are autonomic/automatic forms of applied *reason*; hence, the separation between observation and reason is misplaced. Reason and observation are reciprocally constitutive; memories, for example, influence reason, and reason influences and alters memories (Taleb, 2007). Reason is thus not something which one does; reason is *something which one is.* In this light, obtaining patterns is then *not* a matter of a strong form of *labor*, *expenditure*, *autonomy*, *or creativity*. As argued below, it is the modulation of contexts, which are in a reciprocally constitutive relation with subjects, which enables patterns to emerge. These are known as *discoveries*.

The *active* form of observation, which I define as *assessment* (or meta-assessment, to be more accurate), entails *active selection* and *direct participation*. Meta-assessment would constitute a strong form of labor, expenditure, autonomy, and creativity in the form of *second-order inferences*, as described below. These are considerations of *meta-suitability* or *meta-reasoning*, which are examples of reasoning about the various modalities (and viabilities) of reason itself. In other words, this process involves *biasing certain forms of reason* (and, by extension, the products of reason) over others.

### 4.3 Imagination and an open system

Reasoning is the name given to processes used in abstracting and compressing (and ignoring) information to form sequential narrations. Reasoning is thus a *technique* or the various employed methods of constructing sequences, forming patterns, and collecting data. The various types or techniques of reasoning are called *inferences.* Inferences (like induction, deduction, and abduction) are used to validate, falsify, cast in doubt, maintain, or update sequences or patterns. Hence, reasoning involves constructing *various types of coherences* (different techniques can obtain different patterns or informational content from the same information). Some techniques are more contextually suitable than others.

The applications of these various techniques serve to slice subjective perceptions or experiences (Hegel, 2010; 2018). This process converts each piece (through construction) into spatiotemporal sequential patterns, as functionally usable or functionally relevant data. Reasoning enables the construction of *associative semantic relationships* (a semantic network or semantic web) between observational and/or experiential information with a spatiotemporal location within a subject’s world map. In other words, reasoning creates an *interrelation of dependencies*. Using reason, subjects can construct their own affordances (affordances are advantages or adaptations, which enable and create agency) based on the value and suitability of said data, in a given context. The data’s functional usage has value since it enables the subject to persist (delay the thermodynamic equilibrium of the second law of thermodynamics), maintain, or enhance its viability values and update its world maps (Naidoo, 2023d). Second-order inferences are processes of *repeated meta-assessment*. They are analogous to the Freudian death-drive (Naidoo (2023b). In psychoanalytic terms, this oscillation is known as *hysteria* (Žižek, 2014).

I conceive second-order inferences as those that simultaneously target current and previously obtained data (including memories). These inferences also target the reasoning techniques used to obtain said data, including any data obtained about the catalog of reasoning techniques, thus determining the appropriability of the data and the applied techniques (in terms of maintaining a dynamic equilibrium by ascertaining the viability value of data relating to reason and data relating to obtained data).

Second-order inferences are thus observations about observations or thoughts about thoughts or reason about reason (hence, *meta-reasoning*). These inferences are typically ignored in favor of observed data (Hossenfelder, 2016), especially in the sciences. These kinds of inferences are often labeled as “mere philosophy” in my experience and ignored. However, they are most important since they ensure *that a system remains open and viable*, or, in Popper’s (1945) understanding, a closed system of totalitarianism does not ensure. These inferences are aimed at questioning the natural or accepted order of things and serve to undermine settled positions by demonstrating their inherent contradictions, *a la* Hegel. In other words, transposed into Schrödinger’s (1944) terms, second-order inferences enable a system to persist, avoid their (systems) own entropy increases, and thus avoid thermodynamic equilibrium associated with heat death. Second-order inferences are thus those that maintain a stable-non-equilibrium state, known as a dynamical equilibrium, using *negentropy* (Schrödinger, 1944). In psychoanalytic theory, a closed system is one that has *psychotic* foreclosure, wherein “things” are accepted without question. The goal of psychoanalysis is to move a subject from a state of psychotic foreclosure to a state of hysteria.

Three important questions to answer are as follows: (1) when does data become knowledge? (2) what are the conditions for the acquisition of the status of knowledge (and acquisition of knowledge as such)? and (3) what is learning?

The first two questions are strongly linked and can be dealt with concurrently. Data becomes, or is converted into knowledge *only upon gaining a certain grade of trust*. Trust grading is based on various considerations, such as the invariance (Woodward, 2000) and the value of the data, both of which are related to the aim of maintaining a system’s dynamic equilibrium. Thus, knowledge is data *trusted* to maintain or enhance a system’s dynamic equilibrium. To establish trust, there must be repeated observations and assessments (mainly second-order inferences). The observations and assessments must also target modes of data acquisition/creation, like sampling frequency, error rates, subjective and contextual conditions associated with sampling, the timeframe of the sampling, the sample size, integration with other knowledge, and many others. Trust, like knowledge, is thus *context-sensitive* or relative and needs to be continuously maintained. Both trust and knowledge fluctuate, degrade, or modulate slower in comparison to contextual information and data acquisition. In this way, data and knowledge (as context-dependent constraints) influence and reciprocally constitute one another. Contexts, *as a concept*, I understand as being *the* overarching context-independent constraint. Trust and knowledge exist in a dynamic equilibrium, both serving to maintain an open-dynamic-equilibrium system state. Repeated assessment is necessary, not just repeated observation.

In terms of the truth–knowledge dichotomy, knowledge is *not absolute* (universal or objective) but *relatively-absolute*. Relatively absolute, instead of *absolutely relative,* highlights the distinction between a *postmodern insight* and *insights into postmodernism*. The former would posit that knowledge is completely contextual, which would delight Locke (given his passive mind) and a contextualist like Jacques Derrida. The latter, on the other hand, would suggest that the former replaces one false universal/idol (absolute objectivity or universality) with another false universal/idol (absolute relativity). Thus, the latter attacks the concept of absoluteness*/universality* itself. Hence, absolutely-relative can be explained with reference to art: in societal terms, art cannot be absolute subjectivity or just anything anyone says it is. Art is a singular thing; that singular thing is where *current consensus* lies, particularly in art, and it is situated in an art gallery. In Kantian–Lacanian terms, art, or knowledge, is that which is sublime or imbued with fundamental fantasy (Naidoo, 2023b). The sublime, or the fundamental fantasy, *is trust*. Absolutely relative, as the postmodern, contextualist account of knowledge, typically ignores the underbelly consensus of *unknowledge* or that which is deemed *not trusted*. For example, if I write a paper about the origins of life, which is then disproven by someone else, it may seem like the state of knowledge has not been improved. However, this is not true because invalidation itself serves as a reduction. The state of knowledge *knows what does not describe the origins of life*, which is my invalidated paper.

As pointed out by Popper (1962), the aim of science is not *truth* but rather *knowledge*. Knowledge is conditional; knowledge is only acquired as such through confirmation, repeated assessment, and integration (Woodward, 2000). Science thus aims to build generalized knowledge. Truth is impossible to obtain, argued Popper, because there are infinite paths in history from which knowledge could have originated, making the endeavor of obtaining truth fruitless. Science thus *does not prove; science only confirms through corroboration or refutes*. This means that while knowledge may be relative to space, time (epochs), or spatial–temporal locations, it nonetheless is *relatively absolute* since knowledge is only knowledge as such if it contains *selective trust* (which is an expression of *societal autonomy*). Hegel (2010), anticipating Popper, presented this insight in a different way through his explication of the necessity of contingency. *Facts* are thus forms of knowledge, which have a higher trust value and appear most often in a social context; *but facts are not truth*.

Knowledge and trust are thus based on *consensus*. Consensus is ultimately about building-in, indivisible, irreducible knots into a distributed, networked system (a multi-agent system) (OpenCSF, n.d.). This allows a system to function and maintain a dynamic equilibrium. The issue is that consensus is often interpreted in a closed manner, meaning that it requires the constitution of consensus to be an *agreement* among all participants or agents. However, as voting within politics demonstrates, the presence of a winner does not mean that there is consensus.

Total agreement is unnecessary when the function of consensus, which is irreducibility, is unearthed. In this light, consensus can also be a dis-consensus, meaning a consensus based on a *failure to reach consensus*. This kind of conflict takes the form of “we agree to disagree.” This is an irreducible link, which entangles polemic positions in such a way that it keeps the overall system in an open, dynamic equilibrium (hysteria). Žižek (2008) calls this “oppositional determination,” which, in computer science, is known as “a split brain” in distributed, networked systems (the split brain can be traced back to Kant, who demonstrated the split between understanding and reason. This split was confirmed by Sapolsky (2017) as analogous to the neurobiological workings of the amygdala and the prefrontal cortex. Hegel also described the idea of the split as “unhappy consciousness”). I call this a *pure difference*, which is how Lacan described the way *sexual difference* is articulated in society (Naidoo, 2023b). It is not that there are differences between polar positions or contested points of view; instead, a *meta-difference* is introduced, wherein the *difference itself is conceived differently*. If difference itself is construed differently, there can be *no consensus*, and a dis-consensus results. Dis-consensus ensures that there is a *radical enclosure of openness* within a distributed networked system, enabling the persistence of its status as dynamic, as opposed to static. This is also known as an open society in political terms. In this society, opposing sides remain linked while in a state of continuous observation and assessment because of their very (intentional, unbeknownst to them) oppositional determinations. It enables a system of this sort to continuously and dynamically seek out new gradients of energy or information, which are relevant for viability maintenance. Hence, it is not that identities are used for violence; rather, intentional, conceptual violence is performed *to create identities*. The creation of an indivisible knot as such, which structures a split brain, requires the use of second-order inferences.

On question 3, learning is the process of repeated observation, assessment, validation, correction, storage (memorization), updating, degrading, and relating useful or important semantic, spatiotemporal information within a system’s world map. It is thus an active process intrinsically related to the constitution (construction) or destruction of data, knowledge, and trust. This is the *dual role of imagination*. *Imagination is absent in a closed system*.

### 4.4 Launderer and physicality of information

Ralf Launderer (1961) suggested that information, as a mathematical object, plays a crucial role in physics. His intention was to find the minimum energy required for computation using standard thermodynamics. He used the Launderer reset, which comprises a starting state (say 0) and a binary switch, the latter of which consists of “1” and “0.” Each binary state is a possible logical state for this binary switch. This operation is often referred to as “information erasure” since it reduces the amount of information that can be associated with the binary switch. Before operation, there are two possible states; after operation, there is one. According to thermodynamics, a reduction in the number of possible states for a physical device requires a minimum energy expenditure, which is computable thanks to Boltzmann’s equation. Launderer (1961) then proceeded to deduce the logically irreversible concept, arguing that it implies physical irreversibility. His ultimate deduction was thus that information *must be physical*. However, this spawned much research into logical reversibility, famously by Charles Henry Bennett (1973) and others. Bennet demonstrated that Launderer erred (refer to part B of Supplementary Material).

The claim that information is physical has been refuted, and the current consensus is that it is not physical. An experiment at the NiPS laboratory demonstrated that the logically irreversible gate can be reversed logically with a small amount of energy expenditure (López-Suárez et al., 2016), concluding that there was no fundamental limit and that reversible logic is not required to operate computers with zero energy expenditure. This means that there is no limit as to how much we can lower energy consumption during computation. In turn, this means that information *cannot be physical*; it only has a *physical representation* (Burgin and Mikkilineni, 2022).

Information and physical carriers/representations are different. Different physical carriers can carry the same kinds of information (different brands of pens still carry the information, that it is a pen, used for writing, despite different appearances). Studies like those done by Vopson and Lepadu (2022) demonstrate valuable insights into the teleological movement of information but also have (in conjunction with later studies) *incorrectly described the nature of information* by confusing physical carriers with information. The properties of informational representations and informational carriers are different (Burgin and Mikkilineni, 2022). Information is never directly interacted with; rather, methods of dealing with informational representations and carriers of information are used, like computation, for example. Humans are prone to *conflating* metaphorical symbols with literals due to the recent evolution and organization of the brain, with the prefrontal cortex being an honorary member of the emotional limbic system (Sapolsky, 2017).

### 4.5 Does information have mass?

The current consensus is that information is massless (Burgin and Mikkilineni, 2022). The physical representation of information has mass, which means it would comply with physical laws. This is important to keep in mind.

### 4.6 Place of information

Information forms part of the *world of structures*. In the physical world, entities like genes and neurons process, communicate, and convert information into data (and then knowledge). They communicate information first through a representative analog form, such as biological and neurological structures. Second, communication of such form is achieved with the use of chemical or electrical signals (Burgin and Mikkilineni, 2022). For example, “bits” in the digital world are information, which can have many physically representative forms (like symbols, electrical voltage, or pulses). Information is “carried” by these physical representations in the same way that temperature is “carried” by thermometers (Burgin and Mikkilineni, 2022). The General Theory of Information (GTI) describes and distinguishes the *properties of information* from those of representations and carriers of information.

### 4.7 General theory of information and the physical world

Material structures in the physical world carry information, which represents the *state and the dynamics* of the analog structures mentioned (Burgin and Mikkilineni, 2022). In the physical world, physical or material things are *governed by the transformation laws of matter and energy*; energy can create or change material structures. All physical (including chemical) structures, which are created or changed by the transformation of matter and energy, are governed by and obey transformation laws. Hence, this is how physics distinguishes *what is physical* from *what is not*.

All physical structures contain information, which characterizes their structures, functions of their components (including the interactions of the components with their surroundings), and their behaviors upon the occurrence of fluctuations. Factually, there is a relationship between the characteristics of physical objects, which allows for the conversion of mass into the energy of physical objects described by these characteristics. Einstein’s mass–energy equivalence equation, E = mc^2^, interlinks the energy and mass of physical objects. However, this formula does not mean that substance (matter) is equal to energy; rather, it describes the maximal amount of energy in a physical object with a given mass.

Thus, the states of physical structures and the regularities of their evolution are described by the laws of physics, which are *mental structures created by humans*. “Living” organisms developed physical structures, which exploit matter and energy transformations, to acquire unique identities and the ability to sense and process information, which is carried by material/physical structures. They can do this by converting it into data or knowledge, which are *mental structures*.

All living organisms have varying degrees of perceptive, processing, magnification, and information-to-data-to-knowledge conversion abilities. Humans can typically represent and manage mental structures using *ideal structures or categories* like named sets or *fundamental triads* (Burgin, 2010). Triads provide the schema, including the necessary operations for creating organized forms of data and knowledge, like entities, relationships, and evolutions, based on events and behaviors (Burgin et al., 2020; Mikkilineni, 2022a; Mikkilineni, 2022b). These are world maps.

*Events* are caused by (1) fluctuations in the interactions among the components of structures and (2) fluctuations among components and their niches (Naidoo, 2023d). Function, structure, and fluctuations play important roles in a system’s microscopic and macroscopic behaviors (Prigogine, 1978). Mental models, created by information processing, are *observer-dependent*, as they are conditional on subjective foliations, previous knowledge of the observer, and various other idiosyncratic variables.

### 4.8 General theory of information and the ontological principle

According to this principle, information plays the same role in the world of structures as energy plays in the physical, material world. Despite this link, information is *not part* of the physical world. It can only be materialized in a physical form (Burgin and Krzanowski, 2022; Burgin and Mikkilineni, 2022).

For any portion of information (I), there is always a representation (R) for this (I) in a system. This representation is often material, and because of this, information *seems* physical (Burgin and Mikkilineni, 2022). The physical representation, rather, is the materialization or manifestation of this information and is not the same kind of thing as the information itself. This material form enables the possibility of *social exchange*, given that it allows other subjects to read, process, obtain, and transfer information. DNA is an example of an inanimate transformation and transmittance of information from one physical representation to another. It is the physical/material *representation* of information that complies with physical laws, and not the information itself. Mental processes themselves are also not physical; they are tied to something physical, like the brain, but are themselves not (Davis et al., 2012). In other words, *semantics are not physical*.

In terms of this principle, information in any system can precipitate the potential for, or cause, transformations within the system itself (like changing its structural or logical elements) (Burgin and Krzanowski, 2022).

### 4.9 General theory of information and the representability principle

According to this principle, for any part of information (I), there is always a representation (R) of this part of (I) for a system (S). (R) is a material representation of said information, and it is only (R) only that obeys physical laws.

### 4.10 General theory of information and the embodiment principle

In terms of this principle, for any information (I), there is always also a carrier (C) in a system (S). As a rule, (C) is typically material; hence, (I) is *present* in the material world. (C) is an instance of *materialization* of the information, which I call *the second level materialization sub-principle* (or SLM for short). Consider this example: a piece of paper, as a carrier, requires the materialization of symbolic information (enaction or inscription, in the form of writing letters forming a language) via an instrument, such as a pen. In this example, information is materialized and hence present in the material world when embodied within a carrier, but the materialization in the form of the inked-in written words is only a physical representation of information. The symbols, being the letters of the language, *are also carriers*. This is supported by Shannon and Weaver, who separated the message from semantics.

Thus, any (C) of (I) is a physical something within which (I) is embodied. A physical (R) is also a physical (C) if it allows for the *direct extraction* of the said information. The key difference between (C) and (R) is that any physical representation is a physical carrier, *but not every physical carrier is a physical representation* (Burgin and Mikkilineni, 2022).

To illustrate, consider the following: an envelope is a physical carrier of information (the envelope contains a paper letter with writing on it). The paper letter is also a physical carrier *of the same information* as the envelope since the information embodied within both the envelope, and the paper letter is the text written on the paper. Given that direct extraction is only possible through viewing the text (reading it), it is not possible to extract this text from the envelope without opening it and reading the letter. One also cannot directly extract the text from the paper letter *itself* but *only from the visible writing embodied* on the paper letter. For example, if the letter is written in a visible foreign language, being in possession of the letter does not mean that one can extract the information embodied within it. It is the visible symbols themselves, from which direct abstraction is possible and proceeds (not the paper letter). Hence, the envelope and paper letter within it are only (C). Neither, however, is (R) of the information contained within the letter, since the extraction process cannot be performed on either the paper letter or the envelope. Hence, the difference between FLM (representations) and SLM (carriers) is that FLM comprises SLM, but SLM does not necessarily comprise FLM. A (C) of (I), which is not an (R) (like the envelope or paper letter), is called an enveloping carrier of (I).

The mental worlds of living biological organisms are structured by scaffolding (Naidoo, 2023b). Information obtained from the environment through the senses enables mental representation, which is then converted into mental structures in the form of triads. There are two types of mental structures: (1) those derived from external observations and (2) those created by human minds serving to represent ideal structures. Mathematics is used to represent ideal structures and operators; it is also used to model systems from the material world, their states, and their evolution (Burgin and Mikkilineni, 2022). The mental world/reality contains different mental structures that are involved in transforming information and data into knowledge. These processes are physical processors, namely, genes and neurons.

### 4.11 General theory of information and the rightful placing of information

Information is non-physical (Timpson, 2004; Timpson, 2008; Timpson, 2013), but it is tied to physical and mental structures and processes. Informational (R) and (C) are embodied in other physical and mental structures (Burgin and Mikkilineni, 2022). If the physical (R) is altered, the information changes too. Erasing a representation (like erasing writing)results in (R) losing its status as such since it would no longer embody information. The status as a (C) likewise can be constituted or un-constituted as such.

Symbolic (R) of information is involved in logical or abstract computation (like linguistics), whereas physical computation works with physical (R) and (C) of information (Burgin and Mikkilineni, 2022). GTI locates information not in the world of abstract objects (information exists in things outside of mentality) *but rather within the world of ideal structures. Information appears in mental and physical worlds through materialization and mentalization.* Abstract objects are mental representations of information from the world of ideal structures. They are structures themselves, but they do not belong in the world of ideal structures; they are rather external structures within the general theory of structures.

With regard to living organisms, information can be conceptually (not physically) separated into *ontological information* and *mental information*. The former is that which precipitates formations and transformations of structures within the physical world/physical systems (Burgin and Krzanowski, 2022). Ontological information functions within the physical world; hence, it is used in treating natural phenomena. Mental information (also known as epistemic information), on the other hand, is that which facilitates formations and transformations of structures within the mental world/systems (Burgin and Krzanowski, 2022).

According to GTI, *physical energy* is a type of generalized information situated within the physical world and is that which precipitates the *changing or preserving* of physical systems (Burgin and Krzanowski, 2022). There is a key difference between ontological information and energy as generalized information; the former (as genuine information that can precipitate alterations or preservations of physical systems) acts only on physical systems, which have physical representations, and are embedded in a physical carrier. The latter, energy, *directly acts on physical systems*. Ontological information can also have physical energy as its representation (Burgin and Krzanowski, 2022). This position has also been supported by the demonstration of Maxwell’s demon in laboratories (refer to part B of Supplementary Material) (Hossenfelder, 2016). *Information can be converted into work.* This means that it is possible to replace the transfer of energy from a sender to a receiver with a transfer of information, and this information transfer can occur with much less energy than what the receiver gains from the information (Hossenfelder, 2014).

GTI also distinguishes between mental/epistemic information and mental/psychic energy. Mental/psychic information is generalized information in the mental world that can precipitate the change or preservation of mental systems. Mental information, as epistemic information, is genuine information that can precipitate changes or the preservation of mental systems because of the way it behaves. The difference between both is that mental/psychic information directly acts on mental systems, while mental information, as epistemic information, acts only on systems with a mental representation and embedded within a mental carrier. For example, knowledge is embedded within the mentalities/minds of people. Mental information can have mental energy as its representation (Burgin and Krzanowski, 2022).

## 5 Data governance

Utilizing the framework proposed by Friedrich Hayek (1945) (refer to part C of Supplementary Material), one can answer some of the important questions relating to data governance. The first is ownership of data and the second is the issue of personal data migrations, which bring into play many different, stringent, national and international ethical and regulatory frameworks.

### 5.1 Attribution, not ownership

#### 5.1.1 Objects and order

Imagination is a process of ideation, as I described above, which is the process of constructing and destructing sequential, semantic relations of association. Objects are “crystallized” forms of the imagination, in reference to Erwin Schrödinger (1944) and Ilya Prigogine, 1978. Schrödinger (1944), in *What is Life*, explained that the persistence and resistance of information (moving against thermodynamic equilibrium) are abilities gained from their crystal structures, which keep systems in a dynamic equilibrium. The information is embodied within these solid, physical crystals as patterns/data. Corporeality or solids have shielding properties, enhancing the “stubbornness” of the embodied information. The aperiodicity of solids was fundamental for the evolution of life, as pointed out by Schrödinger (1944). In social systems, humans build houses and take photographs for the same reason.

Imagination is the name of a triadic structure composed of what I call *the big three*. The big three are (1) information, (2) knowledge, and (3) knowhow. Bringing objects from imagination to life requires each of the big three. This often requires assistance from other subjects, such as a structured supply-chain (a distributed network). This is a collaborative effort. A distributed network, as a supply-chain, enables a robust and efficient way to obtain each of the big three and to structure the relations and roles between the different actors of the big three. Each of the big three can originate from different sources. For example, the desire to create an object (a concept)—like a new type of flamethrower—can be my own. The desire and idea are attributable to me; however, I have no *knowledge* of the scientific (or legal) laws and mathematics required to design it, use it, or analyze its possibility of existence. I also do not have the *knowhow*, resources, and technical skills required to bring it into physical manifestation/existence. Of the big three, knowledge and knowhow are the *rarest* (and harder to accumulate). However, knowledge and knowhow are different from the notion of *value*, despite often being equated. The value of products and the value of knowledge and knowhow are *qualitatively different*.

The value associated with products (be it notoriety or economic value) is qualitatively different from the value associated with a person who displays/possesses knowledge sets and knowhow as specific skills, both of which translate into the ability to create products. Both types of value are subject to supply and demand; however, *knowledge and knowhow are applicable to different contexts and different creations/products (higher invariance values)*. In other words, knowledge and knowhow display more invariance because they can directly translate to different contexts and different objects (the knowhow of drilling, for example, can be used to create many different objects). The qualitative difference is that *one value is tied to objects, while the other value is tied to its creator*. Hence, it is the *ability to create* that is most important, *not the creation itself*. That is why the former is afforded privilege, through *attribution*, and requires societal nurturing.

Viewing objects as crystals of imagination explains both their social and economic value. In terms of the former, they enable subjects to *feel* socially linked to one another, thus sharing in a social experience or interrelation. This is the fantasy of equivalency, wherein, through objects, one seemingly feels equivalent to the lived experience of another.

In the latter, statistics relating to imports and exports are important. The objects of import and export are (the exchange of) *crystallized/embodied forms of imagination*. Export structures and statistics reveal information about a country’s ability (and requisite resources) to bring objects from imagination into physical reality. Hence, exports provide information about a country’s knowledge and knowhow. If viewed through the paradigm of crystalized imagination, traditional economic concepts like the balance of trade are ill-suited to their task. An alternative lens is an analysis centered on *balances of imagination*, which involves an imagination exchange (embodied by objects). This also reframes common understandings of exploitation, which are typical in “developing countries” (the idea is that it is exploitative to buy raw materials from a developing country and then sell back to that country an object of higher economic value). The paradigm shift enables linking economic value to the source of imagination, instead of the source of raw materials. Economic prosperity relies on imaginative utility, not on consumption. For example, inventors like Faraday and Tesla developed theoretic frameworks of electromagnetism and methods to make practical applications feasible. These inventors provided imagination for “developing countries” to then see the value of their raw materials. In other words, developing countries are capitalizing on the imagination of others (Hidalgo, 2015).

Hence, economic value is not only understood in terms of the origins of physical order but also includes *the context in which these objects and orders are utilized*. Physical forms of order, or objects, allow for certain functional performances in certain contexts. These functional performances are interrelated with contexts and the arrangements of order embodied as said objects. Arrangement orders precipitate function; the need for different functions precipitates different arrangements.

Medication, for example, is an instance of embodied information, which has greater economic value in some contexts as opposed to others (hence, the economic value is modulated by the contextual conditions). This means that where medicine is produced, *and used*, is important. An instance of medication, being a pill, allows for a deeper description. Intrinsic to the pill’s constitution are the practical uses of the creators’ knowledge, imagination, and knowhow (this is social value). The creators precipitated a disclosure of the potential biological effects of chemical compounds contained in the pill. What is not present in the pill is information relating to how the creators obtained their understanding of these effects and how to synthesize said effects. The practical uses of the pill exist within the context of its use. The development process would have required many resources in the form of the big three. This is the background context, being knowledge of the “what” and the “how” (what connections, rules, and laws were important) and the knowhow, which is the technical skill required to bring it into physical existence (Hidalgo, 2015). It is the background context that renders the pill economically and socially valuable (utility). Contexts also provide for and modulate the value of creators. It is the variety of knowledge and knowhow embodied by various people and objects, which enables better and more creative information processing.

There is *a qualitative difference between practical uses of knowledge, the knowhow embodied within physical objects, and the knowledge and knowhow embodied in people*. The knowledge and knowhow embodied in people are related to the human experience, body, and reality—not the thing (the object) itself*.* It is acquired throughout development in life, scaffold (Naidoo, 2023b) thinking, ideas, and abilities. This is known as “tacit” knowledge, which is a term attributable to Hayek (1945). Tacit knowledge, in my ontology, is descriptive of the type of knowledge that arises due to the *analog form of the human and analog biological structures*, including the brain, neuronal patterns (data), organization of patterns, perception, memories, and experiences. This is the process of *passive observation* (and the content and form of the subjective world map) I detailed above.

#### 5.1.2 Creativity

These patterns, in both subjects and objects, are not subject to *ownership*, but are only *attributable* to persons in terms of said persons being recognized as the rightful creators of said patterns. Here, I briefly discuss the formation and settlement of these ideas, which took place in the early formation of intellectual property (IP) law (refer to part F of Supplementary Material). In the Lockean era, *labor* (as performance) was the source of property and proprietorship. Labor pertains to the *work that went into creating an intangible entity*. The labor concept was flawed, as it did not answer questions related to identifiability; thus, the notion of *creativity* evolved to supplant it.

Creativity describes a specific form of labor, namely, *mental labor*. Mental labor performed by minds enacts processes of creativity, which precipitate in protectable intangibles (in patent law). Although the *common law literary debate* used the *language of identity* more than creativity^1^, I would suggest that creativity described *both the rules and the consequences* of mental performance, whereas the identification aspect added a *surplus rule* (the identifiability of the work with its progenitor). Creativity was an *internal performance*, and identity was the linking of said internal performance of the creator with the created external object.

This understanding of creativity became widespread in the mid-19th century, with leaders such as Thomas Webster (1853), who, in his *Treatise on Designs and Patents*, stated that any products

“of the mind or intellectual labour when embodied in a practical form, whether in books, music, paintings, designs, or inventions in the arts and manufactures’ have the peculiar claim derived from the nature of the subject namely, that the subject matter of such property did not exist like land, the air, or wild-animals … such property is, in the strictest sense of the term, a creation” (Webster, 1853; Burke Inlow, 1950; Sherman and Bently, 2003).

“Creativity,” however, still needed to be described. The understanding was that inventions involved creativity, whereas discoveries were simply *observations of existing natural patterns*, which were not patentable, because this observational process d*id not involve qualitative mental labor, which constituted creation*. The conceptual bridge then to creativity, from discovery, required qualitative mental labor.

Discoveries were understood to be already existing *a priori*, which are context-independent constraints, and always existed independent of human interventions (Webster, 1853; Sherman and Bently, 2003). A genius is one who, as the height of human ability, could ascertain the pool of *a priori*’s, which consisted of scientific laws, ideas, and principles (like gravity or electromagnetism). Like the exclusion of ideas in the literary common law property debate, these *a priori*’s were excluded because of their *universality* (Godson, 1833; Sherman and Bently, 2003). Inventions, however, were objects derived from these *a priori*’s. Inventions were protectable through patent law and *attribution*. Attributions in patent law attributions thus recognize a derived utility from *a priori*’s, arising from creativity. Webster (1853) said

“discoverer is one thing and an inventor is another. The discoverer is one who discloses something which exists in nature, for instance, coal fields, or a property of matter, or a natural principle: such discovery never was and never ought to be the subject of a patent … The Subjects of discovery are indeed sown broadcast; they exist in nature.”

Webster (1853) goes on to note that while there may have been great expenditure in making discoveries, discoveries are not inventions (nor subject to ownership). Instead, discoveries are attributable to other mechanisms, such as awards or recognition. Inventions are those that involve a conversion from *a priori* abstract laws, existing in the minds of men, into something physical and useful. This became the *reduction to practice* requirement in patent law (Sherman and Bently, 2003). Hence, empirical embodiment, drawn from *a priori* concepts and *converted into physical reality*, constitutes an invention. In *Boulton and Watt v Bull* (1795), Justice Buller said that patents “were granted for some production from these elements and not for the elements themselves” (*Boulton and Watt v Bull*, 1795). Thus, the logic of creativity was a *human creation*, and the *object of protection* afforded by patent law was *the human element of creation and creativity in the empirical embodiment of a physical object or process* (Sherman and Bently, 2003). This is the foundation for the *conception* element in patent law, which determines the attribution of data or patterns.

#### 5.1.3 Attribution, not ownership

An important issue to address is the common law ownership of ideas or data (part F of Supplementary Material). The common law ownership of ideas, data, or patterns was explicitly rejected for many legal and social reasons, including the maintenance of a social dynamic equilibrium and an open system. The very constitution of IP as a whole served to close common law property in ideas, data, and the like. The very existence of IP thus serves as a *consensus* regarding the limits of common law protections for legal, viability, and social reasons. IP is thus not something different from the common law of property, but it is *its limit*. IP exists as a context-independent constraint and as a designation for that *which is not* susceptible to common law ownership.

It is important to provide clarity on this position, given that recent works, such as that of Thaldar. et al. (2022), have provided an incorrect and irrational account of the data ownership question within the context of South African law. There are several substantive errors made by Thaldar. et al. (2022), which render their conclusions void. To begin, the relevant question to be answered by the authors is whether data are subject to common law ownership under South African property law. The authors note their methodology/protocol for the article as

“However, *the purpose of this article is not to engage in a normative analysis (what should the law be?), but to engage in a positivist analysis (what is the law?)* that draws attention to the multidimensional legal nature of personal genomic sequence data. Accordingly, we do not develop these normative arguments further in this article” (my emphasis) (Thaldar., et al. 2022).

In exploring the relevant law, the authors note that *physical control* is required as a necessary/peremptory for data to be considered property under the South African common law of ownership. The authors note

“A *potential obstacle to conceiving of data in this way could be the requirement of physical control*, given that personal genomic sequence data are not a corporeal object. It may be recorded on physical devices” (my emphasis) (Thaldar., et al. 2022).

In this light, the authors acknowledge that physical control is a peremptory requirement for data to be considered property under the South African property law. However, in breaking with their methodology, the authors now reject the positivist requirement of physical control and then make a normative argument stating that physical control is outdated. The authors say

“*We suggest that the requirement of physical control is outdated in today’s world where so many valuable assets have a digital rather than a physical existence*, and where these digital assets are effectively controlled via digital device interfaces” (my emphasis) (Thaldar., et al. 2022).

Based on a rejection of a peremptory requirement and thus a positivist analysis as required by the authors’ own methodology, a normative argument is presented so that a conclusion can be constructed that data qualifies as an object under the current South African property law, which is not the case. The conclusion reached by the authors on this matter reflects the following:

“Accordingly, personal genomic sequence data—understood not in the abstract, but as a specific instance of personal genomic sequence data—qualify as property” (Thaldar., et al. 2022).

Had the authors followed their methodology, which is to present the law on the issues of whether data qualify as property under the South African common law of property and thus whether data are susceptible to private ownership, the exact opposite conclusion would have been reached. The rightful and rational conclusion, given the peremptory requirement of physical control, leads to the conclusion that data neither qualify as property nor qualify as being susceptible to private ownership under the South African common law of property.

Additionally, the authors claimed that an instance of personal sequence data is unowned property

“Personal genomic sequence data are *res nullius*—something that belongs to no one” (Thaldar., et al. 2022).

However, the authors have hypostasized data as being property, without meeting the necessary criteria for something to qualify as property. The authors state the following:

“For the purposes of this discussion, the term “property” denotes a legal object (or “thing”) that is susceptible of ownership*. Property can be corporeal (such as a house) or incorporeal (such as intellectual property)”* (my emphasis) (Thaldar., et al. 2022).

However, the authors have made the mistake of conflating corporeality or incorporeality with physicality and non-physicality. For something to qualify as an object of property law, the said object must be *physical*. The authors have made category errors by confusing physical objects, of which corporeality and incorporeality are sub-categories, with non-physical instances such as knowledge, data, or information. Physical objects are objects of the common law, including intangibles. For example, gas is capable of being an object if it is enclosed*; however, qualitatively, gas is categorized as matter, which is both physical and empirical* (Rowlands, 2007)*. Data are not categorized as matter, and that is because data is information or knowledge*, which is the object of intellectual property, and subject to attribution, not ownership.

The objects of both patent law and copyright are data and knowledge (identity in the form of style, in terms of copyright). Both forms exist because of a consensus regarding the societal value of knowledge and the value of the knowhow (the knowledge producer). It is not the physical body of the object that is protected (this is explicitly reserved for the common law); it is the *new knowledge/data* embodied within the object that is protected. This protection is a recognition of the *value of the knowhow producer* through the attribution of legal rights. IP was explicitly created *to govern imagination* (as information, data, knowledge, and knowhow). One of the reasons for this was the perpetuity aspect of the common law, which is harmful to societal equilibrium, and new data/knowledge production. Objects of the common law are physical embodiments of information, not the information, knowledge, or data itself. See part F of the Supplementary Material for more information.

#### 5.1.4 Misunderstanding of physicality and corporeality

Any claim that data can be owned makes the logical error of reification or hypostasis. This is confusing a symbolic pattern with the literal (physical) object. The very first criterion for an object to qualify as such in common law property is *physicality*. I have already described what is considered physical. It is a *category error* to conflate physical objects, of which corporeality and incorporeality are sub-categories, with non-physical instances, such as data, knowledge, or information. Objects can be intangible, incorporeal, corporeal, or fungible and *still be physical*. For example, typically, gas is capable of being an object of the common law only upon its enclosure. Gas, despite its intangible nature, is *qualitatively categorized as matter, which is both physical and empirical* (Rowlands, 2007). This is not to suggest that the premises of the IP law should not be reconsidered.

### 5.2 Distributed, tiered approach

Following Hayek (1945) once more, we can obtain answers regarding the issue of data migrations. It is possible instead to send algorithms to personal/private data storage instead of the other way around. Data produced by the usage of a migrated algorithm would be attributable to the sending party. This potentially limits the risks associated with legal and ethical cross-border data transfers. In terms of data integrity, a Hayekian approach would suggest leaving the decisions to the man on the ground. A solution is simply allowing data analytics companies (any company that performs analytics on datasets. These can be genomic companies, financial companies, legal companies, and so on) to construct their own tiered quality/integrity approach, wherein they charge different amounts depending on a guarantee the company makes related to the quality of the data. Alternatively, data analytics companies can apply the same tiered approach to in-house analytic tools, which can be applied to their in-house datasets. Higher amounts can be charged for higher-quality data or analytic tools. This way, both parties can decide for themselves and rely on contractual provisions and guarantees to regulate their relations.

## 6 Data security and data privacy

**FIGURE 5:**
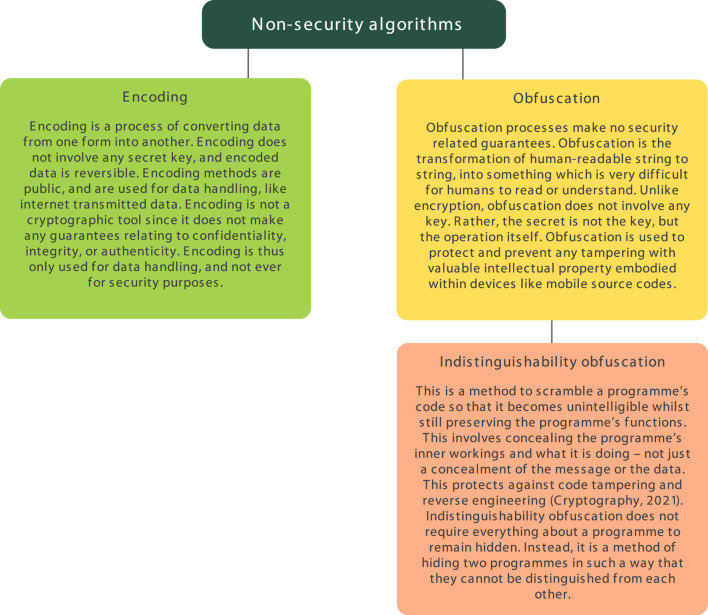
Cryptography. Composed by author, 2023.

**FIGURE 6:**
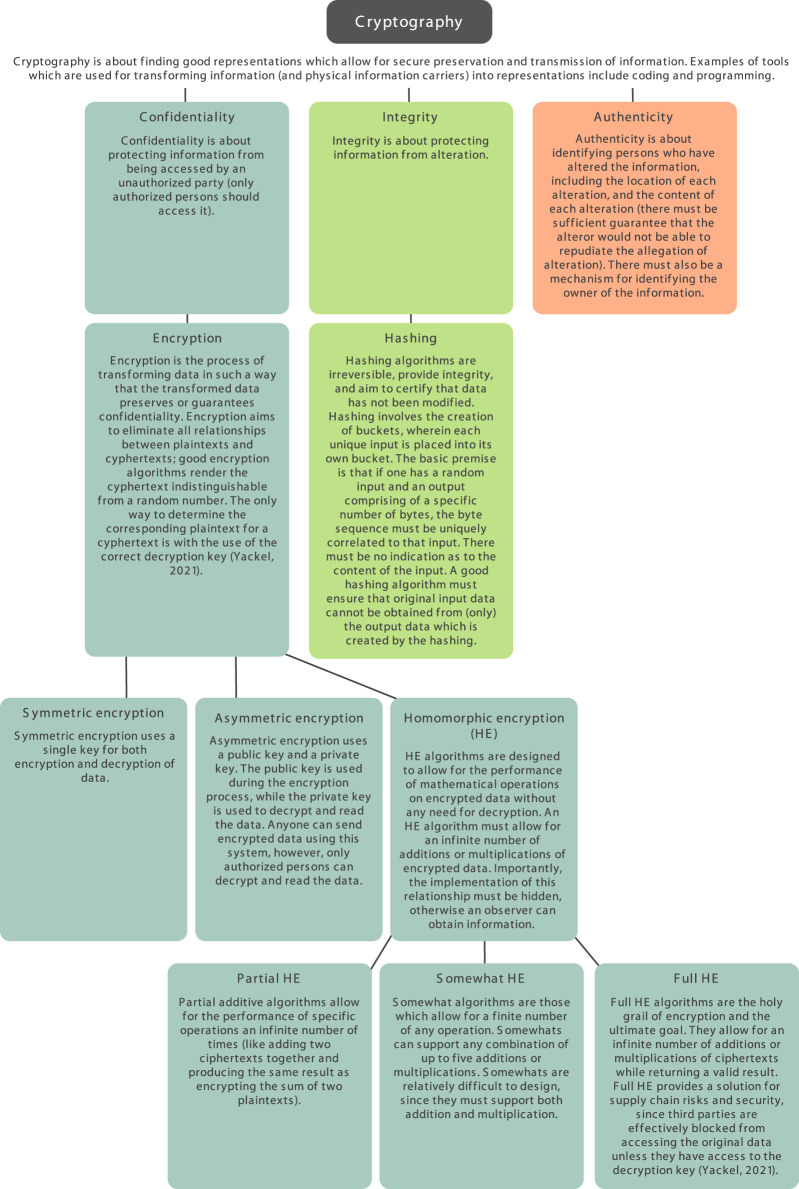
Non-security algorithms. Composed by author, 2023.

**FIGURE 7:**
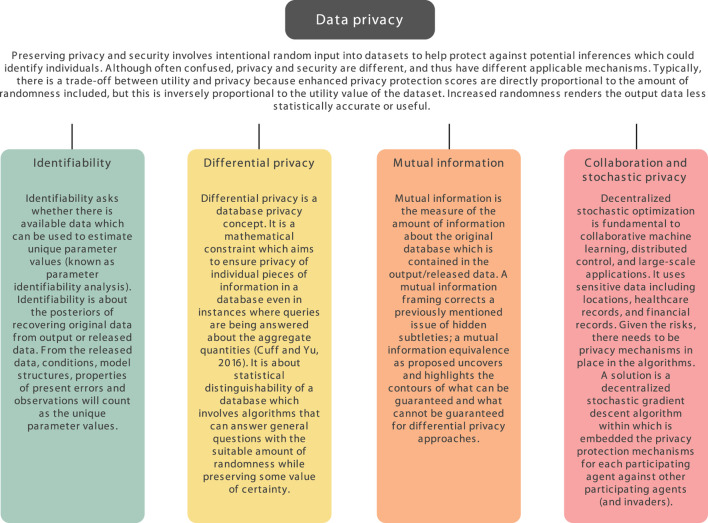
Data privacy. Composed by author, 2023.

Figures 5–7 present serve to add to the ontology. Each figure outlines important concepts for regulators, ethicists, and the private industry to be aware of. For example, often, mechanisms aimed at data security are mixed up with those that are aimed at data privacy (and *vice versa*). The information is introductory, for conceptual clarity and exploration in further work (Figures 5–7).

## 7 Conclusion

Avoiding any lengthy conclusions due to complexity and because I wish any readers of this work to draw their own, I will simply conclude with two things. The first is that I have presented a usable and adaptable framework for an open, dynamically stable, information society, which is applicable to regulatory considerations based on trust and conditionality. Second, I have only provided a negative definition of information. My definition of information is hidden within the first part of this conclusion. Information *is reflection*.

## Data Availability

Publicly available datasets were analyzed in this study. These data can be found at: Naidoo (2023a). An (ontogenetic) transcendental (de)materialist theory of subjectivity. DOI: 10.31219/osf.io/cwugk: https://osf.io/preprints/osf/cwugk. Naidoo (2023b). Contradiction and desire. DOI: 10.31219/osf.io/up5f4: https://osf.io/preprints/osf/up5f4. Naidoo (2023c). The hard problem, qualia, agency, intelligence, and Freud. DOI: 10.31219/osf.io/gqspk: https://osf.io/preprints/osf/gqspk. Naidoo (2023d). What does it mean to be an Agent? DOI: 10.31219/osf.io/evna6: https://osf.io/preprints/osf/evna6 (now published on Frontiers at https://www.frontiersin.org/journals/psychology/articles/10.3389/fpsyg.2023.1273470/full).

## Footnotes

1 There are finer distinction and details, but they do have much in common as they are both performance-based.

## References

[B1] Althusser L. (2014). On the reproduction of capitalism: ideology and ideological state apparatuses. London: Verso.

[B2] Badiou A. (2006). Logics of worlds: being and event II. Continuum.

[B3] Bennett C. H. (1973). Logical reversibility of computation. IBM J. Res. Dev. 17 (6), 525–532. 10.1147/rd.176.0525

[B4] Benveniste E. (1966). Problems in general linguistics. Chicago: University of Chicago Press.

[B5] Bernstein M. N. Castellano G. (2020). Information entropy. Found. Inf. theory Part 2, 249–257. 10.1201/9780429022937-23

[B6] Boulton and Watt v Bull (1795). 126 er 662.

[B7] Brownlee J. (2019). A gentle introduction to information entropy. Machine Learning Mastery. Available at: https://machinelearningmastery.com/what-is-information-entropy/ (Accessed August 2, 2023).

[B8] Burgin M. (2010). Theory of information: fundamentality, diversity, and unification. World Scientific Publishing Company.

[B9] Burgin M. Krzanowski H. R. (2022). World structuration and ontological information. Proceedings 81 (1), 1–4. 10.3390/proceedings2022081093

[B10] Burgin M. Mikkilineni R. (2022). Is information physical and does it have mass? Information 13 (11), 1–10. 10.3390/info13110540

[B11] Burgin M. Mikkilineni R. Phalke V. (2020). Autopoietic computing systems and triadic automata: the theory and practice. Adv. Comput. Commun. 1, 16–35. 10.26855/acc.2020.12.003

[B12] Burke Inlow E. (1950). The patent grant. Baltimore: The Johns Hopkins Press.

[B13] Chalmers D. (1996). The conscious mind: in search of a fundamental theory. Oxford: Oxford University Press.

[B14] Chalmers D. J. (2010). The character of consciousness. Oxford: Oxford University Press.

[B15] Cryptography (2021). What is indistinguishability obfuscation? Available at: https://crypto.stackexchange.com/questions/44770/what-is-indistinguishability-obfuscation (Accessed July 11, 2023).

[B16] Cuff P. Yu L. (2016). Differential privacy as a mutual information constraint. 10.1145/2976749.2978308

[B17] Damasio A. (1991). Somatic markers and the guidance of behavior. Oxford: Oxford University Press.

[B18] Damasio A. (2005). Descartes’ error: emotion, reason, and the human brain. New York: Penguin Books.

[B19] Davis S. F. Palladino J. J. Christopherson K. (2012). Psychology. London: Pearson.

[B20] Dawkins R. (1976). The selfish gene. Oxford: Oxford University Press.

[B21] Deacon T. W. (2007). Shannon – Boltzmann — Darwin: redefining information (Part I). Cogn. Semiot. 1, 123–148. 10.1515/cogsem.2007.1.fall2007.123

[B22] Descartes R. (1641). *Meditations on first philosophy* (translated by elizabeth S. Haldane).

[B23] Deutsch D. (1998). The fabric of reality. London: Penguin Books.

[B24] Di Giovanni G. Livieri P. (2018). Friedrich Heinrich Jacobi. Available at: https://plato.stanford.edu/entries/friedrich-jacobi/ (Accessed October 14, 2022).

[B25] Dimitrov B. G. (2015). Topological (in) Hegel: topological notions of qualitative quantity and multiplicity in Hegel’s fourfold of infinities. PhD thesis, University St. Kliment Ohridski).

[B26] Dolar M. (1991). I shall Be with you on your wedding-night: lacan and the uncanny. Lacan uncanny 58, 5–23. 10.2307/778795

[B27] Einstein A. Podolsky B B. Rosen N. (1935). Can quantum-mechanical description of physical reality be considered complete? Phys. Rev. 47, 777–780. 10.1103/physrev.47.777

[B28] Floridi L. (2009). “Philosophical conceptions of information,” in Formal theories of information: from Shannon to semantic information theory and general concepts of information muenchenwiler seminar (Switzerland). 10.1007/978-3-642-00659-3_2

[B29] Freud S. (1915). *The Interpretation of Dreams* (translated by brill, A. A.). The Macmillan Company.

[B30] Godson R. (1833). Law of patents. *Hansard col* , 15.

[B31] Hartnett K. (2022). How Shannon entropy imposes fundamental limits on communication. Quanta Mag. Available at: https://www.quantamagazine.org/how-claude-shannons-concept-of-entropy-quantifies-information-20220906/ (Accessed January 18, 2023).

[B32] Hawkins J. Dawkins R. (2021). A thousand brains: a new theory of intelligence. New York: Basic Books.

[B33] Hayek F. A. (1945). The use of knowledge in society. he American Economic Review 35 (4), 519–530. http://www.jstor.org/stable/1809376.

[B34] Hegel G. W. F. (2010). *Science of logic* (translated by G. Di Giovanni). Cambridge: Cambridge University Press.

[B35] Hegel G. W. F. (2018). *Phenomenology of Spirit* (translated by T. Pinkard). Oxford: Oxford University Press.

[B36] Hidalgo C. (2015). Why information grows: the evolution of order, from atoms to economies. New York: Basic Books.

[B37] Horkheimer M. Adorno T. W. (1989). Dialectic of enlightenment. New York: The Continuum Publishing Company.

[B38] Hossenfelder S. (2014). The remote Maxwell demon. Back ReAction. arXiv. 10.48550/arXiv.1408.3797

[B39] Hossenfelder S. (2016). The remote Maxwell demon as energy down-converter. Found. Phys. 46, 505–516. 10.1007/s10701-015-9981-7

[B40] Juarrero A. (2023). Context changes everything: how constraints create coherence. Cambridge: MIT Press.

[B41] Lacan J. (1979). *The Seminar of Jacques lacan, book XI: the four fundamental Concepts of psychoanalysis* (translated by A. Sheridan). W. W. Norton and Company.

[B42] Landauer R. (1961). Irreversibility and heat generation in the computing process. IBM J. Res. Dev. 5 (3), 183–191. 10.1147/rd.53.0183

[B43] Lock J. (1860). An Essay concerning human understanding. Available at: http://www.philotextes.info/spip/IMG/pdf/essay_concerning_human_understanding.pdf (Accessed January 18, 2023).

[B44] López-Suárez M. Neri I. Gammaitoni L. (2016). Sub-kBT micro-electromechanical irreversible logic gate. Nat. Commun. 12068 (7), 1–6. 10.1038/ncomms12068 PMC4931245 27350333

[B45] Marx K. Engels F. (1848). “Manifesto of the communist party,” in Marx/engels selected works. Editors Marx K. Engels F. (Moscow: Progress Publishers), 98–137.

[B46] Mikkilineni R. (2022a). A new class of autopoietic and cognitive machines. Information 13, 24–14. 10.3390/info13010024

[B47] Mikkilineni R. (2022b). Infusing autopoietic and cognitive behaviors into digital automata to improve their sentience, resilience, and intelligence. Big Data Cogn. Comput. 6, 7–15. 10.3390/bdcc6010007

[B48] Mokyr J. (2012). The enlightened economy: an economic history of britain 1700-1850. New Haven: Yale University Press.

[B49] Naidoo M. (2023a). An (ontogenetic)transcendental (de)materialist theory of subjectivity. 10.31219/osf.io/cwugk

[B50] Naidoo M. (2023b). Contradiction and desire. 10.31219/osf.io/up5f4

[B51] Naidoo M. (2023c). The hard problem, qualia, agency, intelligence, and Freud. 10.31219/osf.io/gqspk

[B52] Naidoo M. (2023d). What does it mean to be an Agent? 10.31219/osf.io/evna6

[B53] OpenCSF (n.d.). Consensus in distributed systems. Available at: https://w3.cs.jmu.edu/kirkpams/OpenCSF/Books/csf/html/DistConsensus.html (Accessed May 17, 2023).

[B54] Plato (2002). The republic. Available at: https://www.sciencetheearth.com/uploads/2/4/6/5/24658156/plato_-_the_republic.pdf (Accessed May 17, 2023).

[B55] Poincaré H. (2010). *Papers on Topology: analysis Situs and its five Supplements* (translated by J. Stillwell). American Mathematical Society.

[B56] Popper K. (1945). The open society and its enemies. London: Routledge.

[B57] Popper K. R. (1962). On the sources of knowledge and of ignorance. Philosophy Phenomenological Res. 23 (2), 292–293. 10.2307/2104935

[B58] Prigogine I. (1978). Time, structure, and fluctuations. Science 201 (4358), 777–785. 10.1126/science.201.4358.777 17738519

[B59] Rosen W. (2010). The most powerful idea in the world: a story of steam, industry, and invention. London: Jonathan Cape.

[B60] Rowlands P. (2007). Zero to infinity: the foundations of physics. World Scientific Publishing Company.

[B61] Sapolsky R. M. (2017). Behave: the biology of humans at our best and worst. New York: Penguin Books.

[B62] Schrödinger E. (1944). What is Life? Cambridge: Cambridge University Press.

[B63] Schrödinger E. (1967). What is life? The physical aspect of the living cell with mind and matter & autobiographical sketches. Cambridge: Cambridge University Press.

[B64] Shannon C. E. (1940). A symbolic analysis of relay and switching circuits PhD Thesis (Massachusetts Institute of Technology). Available at: https://dspace.mit.edu/handle/1721.1/11173 .

[B65] Shannon C. E. Weaver W. (1964). The mathematical theory of communication. Illinois: The University of Illinois Press.

[B66] Sherman B. Bently L. (2003). The making of modern intellectual property law: the British experience, 1760–1911. Cambridge: Cambridge University Press.

[B67] Spinoza B. (1995). *Spinoza: the letters* (translated by S. Shirley). Indianapolis: Hackett Publishing Company.

[B68] Sproll P. A. C. (1994). Matters of taste and matters of commerce: British government intervention in art education in 1835. Stud. Art Educ. 35 (2), 105–113. 10.2307/1320824

[B69] Taleb N. N. (2007). The black swan: the impact of the highly improbable. New York: Penguin Books.

[B70] Thaldar D. (2017). The open society: what does it really mean? De. Jure 50 (2), 396–405. 10.17159/2225-7160/2017/v50n2a11

[B71] Thaldar D. W. Townsend B. A. Donnelly D. L. Botes M. Gooden A. van Harmelen J. (2022). The multidimensional legal nature of personal genomic sequence data: a South African perspective. Front. Genet. 13, 997595. 10.3389/fgene.2022.997595 36437942 PMC9681828

[B72] Timpson C. (2004). Quantum information theory and the foundations of quantum mechanics. Oxford: University of Oxford. [PhD thesis].

[B73] Timpson C. (2008). “Philosophical aspects of quantum information theory,” in The ashgate companion to the new philosophy of physics. Editor Rickles D. (London: Routledge), 197–261.

[B74] Timpson C. (2013). Quantum information theory and the foundations of quantum mechanics. Oxford: Oxford University Press.

[B75] Vopson M. Lepadatu S. (2022). Second law of information dynamics. AIP Adv. 12, 1–7. 10.1063/5.0100358

[B76] Webster T. (1853). On property in designs and inventions in the arts and manufactures. Chapman & Hall.

[B77] Wiener N. (1961). Cybernetics: or the control and communication in the animal and the machine. 2 ed. Cambridge: MIT Press.

[B78] Woodward J. (2000). Explanation and invariance in the special sciences. Brit. J. Phil. Sci. 51, 197–254. 10.1093/bjps/51.2.197

[B79] Yackel R. (2021). What is homomorphic encryption, and why isn’t it mainstream? Key Factor. Available at: https://www.keyfactor.com/blog/what-is-homomorphic-encryption/ (Accessed May 4, 2023).

[B80] Yoshida R. (2010). Self-oscillating gels driven by the Belousov-Zhabotinsky reaction as novel smart materials. Adv. Mater. 22 (31), 3463–3483. 10.1002/adma.200904075 20503208

[B81] Žižek S. (2003). The puppet and the dwarf: the perverse core of christianity. Cambridge: MIT Press.

[B82] Žižek S. (2008). The ticklish subject: the absent centre of political ontology. London: Verso.

[B83] Žižek S. (2014). *The most Sublime hysteric: Hegel with lacan* (translated by T. Scott-railton). Cambridge: Polity Press.

