# Supplementary material for: The open ontology and information society
Source: Front Genet. 2024 May 30;15:1290658. doi: 10.3389/fgene.2024.1290658 (PMC11170149; doi:10.3389/fgene.2024.1290658)
Supplement: Supplementary file 1 [file DataSheet4.PDF]

Supplementary Material for

The open ontology and information society

1 Part A: The vanishing mediator and society

1.1 The Vanishing mediator: Capitalism (modern science) and religion

The Protestant Reformation which took place in 16th century Europe, was a movement within Western Christianity which successfully challenged the spiritual and political power of the Roman Catholic Church (Zucker and Harris, n.d.). The Reformation emphasized that individuals should be able to read the Christianity's Holy Scripture for themselves without the assistance of the Church, as an institution, or Priests, as mediators. Luther, an Augustine monk, discovered texts which formed the basis of the Reformation principle "sola gratia"—man's salvation comes by God's grace alone and does not in any way depend on his works—which went against the ideas that the Catholic Church had imposed, which resulted in clash for power between the Catholic Church and the Protestants. The Catholic Church argued that without a proper interpretation of the Scripture, the commoners (the majority of whom could neither read, nor had access to the Bible was restricted by the fact that all copies were handwritten, limited, and expensive) would not be able to correctly interpret the word of God. Luther however contended that meaning comes from the readers interpretation of the Bible, and not the Clergy. This does not mean that Priests, and those of the Church should no longer be able to assist commoners should they request it (nor should their historical interpretations be discarded). The Protestants simply argued that the Bible should be accessible by all in their own languages, and for them to interpret for themselves. This led to individual empowerment and liberation from the morality and values that the Church prescribed. Commoners were able to develop their own diversification of faith, with different sects, that lead to whole new corpus of values, interpretations, and culture. Calvinism as being the major branch of Protestantism (the theology of John Calvin which drew from the work of Augustine of Hippo) (Bouwsma, 2020) consists of a defined ethic including that of working hard, individual drive and due returns ("bootstrapping").

Fredrich Jameson (1973) was the first to coin the phrase "vanishing mediator". This term describes an event, or a thing, which sets the structure and common understanding for existence, reality, or social systems themselves. In other words, it is a something which forms the scaffolding for something, then vanishes into, and as part of the content of that which is scaffolds. This occurs in biology, as Moreno and Mossio (2015) point out, where external boundaries can become internal ones (like the move from invertebrates to vertebrates, which is known as *shanghaai'ing*). It is thus a mediator, which mediates, then "vanishes". What does it mean to vanish? The mediator vanishes *only conceptually*, because it becomes *absolutely universalized to the point wherein one cannot see/distinguish it anymore*. It is like Stockholm syndrome, where traumatized individuals lose their separation between trauma and love.

The Calvinistic ethic is another good example; the ethic set the structures for capitalism (namely, how individuals see themselves in relation to the world). *The Calvanistic ethic is the vanishing mediator for both capitalism and liberal democracies today*. For Jameson, secularism is not the inclusion/exclusion of religion. It is also not that that religious life becomes more secular. What happened was that *secular life became so religious, that it no longer considers itself religious*. That is capitalism.

The system contains the Calvinist and Protestant ethic, which has been universalized to the degree that its status as the initial catalyst fades away. Persons in society, do not experience their capitalist life as being Calvanist, because every aspect of their lives are imbued and shaped by the ethic. In other words, *religious life becomes universalized in capitalism, and it is religion that fades away as the catalyst*. Other thinkers like Thomas Frank and Walter Benjamin have noted that that the attachments/participation to a specific type of capitalistic framework, does not have anything to do with any Enlightenment justifications (like empirical or rational justifications); instead, it has to do with *a religious attachment* to the *profit motive instead*. *Capitalism exists as a form of disavowed religion—which is religion that is both universalized and emptied out of its former positive content*.

1.2 Modern Science and Religion are the same thing!

The issue of constraints is a massive issue in modern physics. The field is mature, with many effective theories applicable to different contexts, by observation and experimentation. Hence, these theories have now become vanishing mediators themselves, forming axioms or scaffolds for the progress of science and research. New theories must fit these axioms, if they want to be considered. If not, they will not be considered or funded. *New experiments are hence constrained by old ones* (Hossenfelder, 2018). These constraints, as axioms, are in fact so strong that it is near impossible to construct new experiments and theories today, because they rule out novelty, even if that novelty can produce incredible results. In other words, it cannot be new, if it does not fit in with the old. *This is how physicists determine normatively what is right and what is wrong*. *The type of beauty found or aimed for in modern science is that of rigidity* (Hossenfelder, 2018). Unfortunately, these attachments, only serve to constrain *meta-freedom*.

Science was in fact referred to, and the embodiment of the marketplace of ideas (Hossenfelder, 2018). The practice was supposed to be about testing, validating and imbuing knowledge as such, with trust; but science does not seem to be self-reflective. Luckly, big names in physics like Sir Roger Penrose and Sabine Hossenfelder have acted as the conscience, or second-order reason, of science. There needs to be a scientific method for the non-physical. No longer, should science just confine itself, with physical objects, in the same way that the foundations for relations within society need to change. Science practice does not cater to everyone, just other scientists. Scientific experts judge one another's products; with success being determined by the products ability to explain an observation. Before that, however, it must be approved by peers (Hossenfelder, 2018). This approval determines whether theories would even be tested.

Within the common marketplace idea, is that the value of goods is determined by the market. The value of scientific explanations is determined by the explanations ability to fit observations. This observational value is unknown at the point of starting one's research. Science therefore is not a marketplace which creates its own value; instead, it is a forecasting platform to identify an external value. The scientific community, first, must select the most promising ideas and then will they support

them. This means that there are already distortions at play before theories can breathe, hence the marketplace does not work (Hossenfelder, 2018). In this way, experimentation is a stranglehold for new theories and ideas, prior to even being considered, let alone performed.

Unfortunately, this is the result of the Vienna circle. The Vienna circle was the culmination of scientific empiricism; but a poor version of it, which mixed the perils of favoritism and capitalism into a broth. This broth included its own version of chemical (X), which is "pretending". The pretense, which will be dealt with in further work, was that some of the scientific ideas, or revolutions, were the work of said scientists, and not the philosophers who came before. The most cherished members of the circle included Einstein, who reigned supreme over all others (Uebel, 2020). As I have learned in my business endeavors, *investors invest in people, and not the product.* Thus, even though theories may be flawed; the *geists* of dead men remain *hypostasized because of the investment in the person, and not the suitability of the idea itself*.

1.3 The missing political center

The anthropologist Claude Levi-Strauss (2011) (often called the founder of structuralist anthropology) discussed social inequality with reference to a tribe, which was split into two camps. Camp one, when asked to describe their society, drew a map which *presenting an unequal society*.

Camp two on the other hand, when asked to do the same, draws a map of an equal society. Neither are correct, *since they are not comparable* in Extremistan systems (Taleb, 2007). However, the insight is valuable. The marginalized (less powerful, Camp two) communities main societal framing device is that of *inequality*. The other, more powerful camp, Camp one, present equality and centrism as being their prime societal framing device. Both framing devices are distortions, *based on different Master Signifiers* (different evaluative criteria). The right (Camp two) uses the *false universal of equality and centrism. The other less powerful camp, use the false universal of inequality and class conflict.*

The centrist position of Camp one, presents the *golden mean* as the center, from which everything outside said center, is a deviation in equality (either left or right). The center is thus the starting point for Camp A. Camp B on the other hand, describes society as being either left, or right, with the center, as the golden mean, being posited retroactively/in retrospect, instead of at the start. Hence, the center for both, although coming at different stages, is the false universal. It is a golden mean/average, which is fundamentally not possible (Taleb, 2007). With camp one, we begin with the center, then deviate left or right, from said center. Camp two, the center does not exist at the start at all; the center only emerges retroactively/retrospectively as the manifestation of the inequality between the different camps, which emerges as a disavowal of said inequality. Another false universal. When one critiques society, and argues that the centrist position posited by the other is false, *one is already situated within the inequality framing* (which retroactively posits the center). *The larger political struggle is the question of – what does the center actually mean?* Do we have an a priori center from which we get deviations, or do we have antagonisms (like social struggles) which then retroactively create the illusion of the center?

Žižek's (2008) insight, is that *the truth* of society is *not centrism*; the truth of society is *the misrecognition* of there being a center at all. Thus, the truth of society is neither equality nor inequality, but rather just misrecognition and difference. Both opposing sides create one another. This is called "oppositional determination." This is exemplar of what Locke called madmen; people who reason correctly from erroneous premises. Thus, what defines a *healthy society,* is the very

impossibility or inability to decide on what a healthy society or its grounding is. This is dis-consensus, as the irreducible link.

1.3.1 The Gaussian/Bell-curve in social matters and physics

This is a non-scalable system. Variables which belong to a system of this sort include things like height, weight, car accidents, income, morality rates and IQ (Taleb, 2007). When Gauss developed the Gaussian method, he intended for it to be used to measure massive errors in distribution; it was called the *la loi des erreurs* (Taleb, 2007) (the law of errors). Any divergences from the mean were errors. Although Gauss came up with the method, the popularizers who developed the notion of the bell curve and its applicability in different fields and domains were gambler called *Abraham de Moivre* and *Adolphe Quételet* (Taleb, 2007). The latter came up with the notion of the reasonable man/average human (*l'homme moyen*). Quételet decided that he wanted the world/reality to fit his average; this average was to be understand as the 'normal'. His Bell curve endeavors were supported and fed by the times too; this was around the time of Marx (Taleb, 2007) and Saint-Simon and the rise of strains of socialism. In this epoch, these thinkers were seeking the "golden mean", some sense of averages in physical attributes, wealth and the rest. So Quételet began working with statistics on different physical things – and created standards for each of them. Deviations from his norm were rare – and increasingly rarer as the magnitude of the deviation increased (as described). After modelling the physical characteristics of humans (the average human), he focused on doing same for social matters, namely things like consumption, manners of life, morality, and habits. There was a *l'homme moyen* for each of these variables. He created a range of deviation from the average (both left and right from the center). Those deviations in the extreme left or right are "ignorable" and, in fact, "abnormal". His system was purely quantitative (based on specific attributes which can vary significantly).

The averageness doctrine is illogical. Parameters can be only parameters if there is difference which creates them. In other words, there is no person who could be average in every single parameter. As Taleb (2007) states, the average human would be half male, half female. Nonetheless, the notion of the average man was attractive to thinkers like Quételet and Marx because of the context of the times in which they were in. The misuse of the Gaussian is the source of the incorrect notion of wealth distribution and deviation; and it is also the source (within policy making) of confusing the "is" with the "ought". The Gaussian nonetheless is used and attractive only because it makes things *appear less random* (Naidoo, 2023d).

In physics there is what is known as the paradox of the infinite divisibility of time. One can think back to the race between Achilles and the Tortoise. Achilles would never be able to catch up the Tortoise to whom he has given the lead. This is because every time he thinks that he has caught up, the tortoise has also moved slightly ahead of him. In other words, the previous point he was trying to catch up to, no longer existed since the tortoise moved too. If he catches up to the new point of the tortoise, the same thing happens. What if he then passes the tortoise? He still has not caught up because that point has changed again. The Tortoise is the frame of reference for the observer, not Achilles. This is also an instance of preferential attachment or cumulative advantage (below). Achilles never catches the tortoise because the race cannot be divided into finite intervals. Fundamentally, time cannot be divided (into instances) at all (Rowlands, 2007). Space however can!

2 Part B: Information

Norbert Wiener (1961) provided a *negative* (exclusionary) definition of information in *Cybernetics*. Wiener (1961) said that:

> "As a final remark, let me point out that a large computing machine, whether in the form of mechanical or electric apparatus or in the form of the brain itself, uses up a considerable amount of power, all of which is wasted and dissipated in heat. The blood leaving the brain is a fraction of a degree warmer than that entering it. No other computing machine approaches the economy of energy of the brain. In a large apparatus like the Eniac or Edvac, the filaments of the tubes consume a quantity of energy which may well be measured in kilowatts, and unless adequate ventilating and cooling apparatus is provided, the system will suffer from what is the mechanical equivalent of pyrexia, until the constants of the machine are radically changed by the heat, and its performance breaks down. Nevertheless, the energy spent per individual operation is almost vanishingly small, and does not even begin to form an adequate measure of the performance of the apparatus. The mechanical brain does not secrete thought 'as the liver does bile,' as the earlier materialists claimed, nor does it put it out in the form of energy, as the muscle puts out its activity. Information is information, not matter or energy. No materialism which does not admit this can survive at the present day".

2.1 Shannon information theory

Information theory is used to provide measures for comparing coding schemes of parts, and their ensembles (wholes). Information theory *aims to avoid biases* caused by preconceptions of codes and complications, which are inherent to non-linear systems with complex interactions.

In his endeavor to create optimization, Shannon had to deal with qualitative and quantitative aspects of information (in its abstract form) itself (Shannon and Weaver, 1964). In the work, Shannon noted the following:

> "Frequently the messages have *meaning*; that is they refer to or are correlated according to some system with certain physical or conceptual entities. These semantic aspects of communication are irrelevant to the engineering problem. The significant aspect is that the actual message is one selected from a set of possible messages. The system must be designed to operate for each possible selection, not just the one which will actually be chosen since this is unknown at the time of design" (Shannon and Weaver, 1964).

In the same work, Weaver says of information:

> "The word information, in this theory, is used in a special sense that must not be confused with its ordinary usage. In particular, information must not be confused with meaning" (Shannon and Weaver, 1964).

Continuing, Shannon says the following:

> "The fundamental problem of communication is that of reproducing in one point either exactly or approximately a message selected at another point. Frequently, the messages have meaning…These semantic aspects of communication [referring to the meaning of a message] are irrelevant to the engineering problem" (Shannon and Weaver, 1964).

2.2 Launderer and Bennet

On Logical irreversibility, Launder (1961) said –

> "We shall call a device logically irreversible if the output of a device does not uniquely define the inputs. We believe that devices exhibiting logical irreversibility are essential to computing. Logical irreversibility, we believe, in turn implies physical irreversibility, and the latter is accompanied by dissipative effects".

Launderer (1961) also said:

> "In fact, most of the standard logic operations in ordinary computers show 'logical irreversibility.' This is the case, for example, of the 'OR' gate, in which there are two bits at the input and one bit at the output. In this way, the sole knowledge of the value of the output is not enough to infer the actual values of the inputs (from this the idea of 'irreversibility')".

From this, Launderer (1961) deduced that *information then must be physical*. Responding to Launderer, Bennet (1973) said –

> "Landauer has posed the question of whether logical irreversibility is an unavoidable feature of useful computers, arguing that it is, and has demonstrated the physical and philosophical importance of this question by showing that whenever a physical computer throws away information about its previous state it must generate a corresponding amount of entropy. Therefore, a computer must dissipate at least kBT ln2 of energy (about 3 X 10-21 Joule at room temperature) for each bit of information it erases or otherwise throws away".

2.3 Maxwells demon, information, and electricity

Maxwells demon is a thought experiment, by James Maxwell (1867), which hypothetically "violates" the second law of thermodynamics. The experiment consisted of a demon which controls a small massless door between two chambers of gas. Each chamber is filled with a mix of fast-moving and slow-moving molecules. When individual gas molecules or atoms move towards the door, the demon opens quickly and allows only the fast-moving ones to pass through in one direction, and the slower moving ones to pass through in the other direction. The kinetic temperatures of gases depend on the velocities of its constituent molecules—the actions of the demon cause one chamber to warm up and the other to cool down. *This results in a total entropy decrease of the system without applying work (energy exchange)*—hence violating the second law.

However, this does not actually violate the second law. It has been demonstrated that for the demon to store, maintain, and use information, it must either give up its own low entropy state or it must have access to an energy source (Hossenfelder, 2016). This means that it must start with a low entropy which then increases, or the demon has an infinite reservoir of low entropy. Thus, the total entropy never decreases, and the second law is not violated (Hossenfelder, 2014). What happens is that the demon uses information to drive a system out of equilibrium, which allows the system to then do work.

2.4 Entropy

There are two paradigms in which to view entropy: (1) a measure of uniformness and (2) as the best achievable rate of compression. In the first paradigm, entropy is the degree of uniformness of (X); the higher the entropy of (X) the closer (X) is to having all outcomes being equiprobable. Entropy is maximal where the probabilities are uniformly distributed over outcomes (equiprobable). This is a

state of lack-of-order, static equilibrium, heat death, or a closed, unhealthy society *a-la* Popper (1945) and Althusser (2014).

There is correspondence between the anticipated degree of surprise obtained from the outcome of (X), and how random/uniform (X) itself is. In instances where we know the certain outcome, the entropy is 0 (zero) (and low information gain). When outcomes are all equally likely, then there is more surprise, and there would be a high entropy attached to a uniform (X) (and more information gain). Thus, in instances where (X) has a high entropy, it is closer to being a uniform variable; in instances where (X) has a low entropy, it is less uniform because there is a high probability of occurrence for a lesser amount of its outcomes (some outcomes a more probable than others).

2.5 Bayes theorem

Bayes law/theorem states that A and B are descriptive of events. P(A|B) describes the probability of A, if B is taken as being true. This is *conditional probability*, or posterior probability of A given B. What would be the probability of A occurring if B is true. P(B|A) on the other hand is descriptive of the probability of B if A is given as true (an exact reverse). P(A), P(B) is descriptive of the independent probabilities of A and B (Byjus, (n.d.)).

In other words, Bayes law describes the probability of an event occurring based on prior knowledge of conditions which may be related to said event. Bayesian inference describes a method of inferential reasoning wherein a *degree of belief* (expressed as a probability) is assessed, and its suitability or necessity to change said belief (update) is determined with reference to new contextual evidence. This kind of method thus evaluates continuous contextual evidence, to produce an outcome of a *reduction of uncertainty, and surprise* (DeWeese and Meister, 1999). Information gain is thus tied to uncertainty, and continual updates. Bayesian surprise is the name for the quantification of said information gain (Naidoo, 2023d).

3 Part C: Friedrich Hayek

3.1 Disorganized-organized knowledge

Friedrich Hayek (1945) and the Austrian school of thought were the first to come up with a proper critique and formulation of financial and economic theory of knowledge, which still exists today, forming the basis of modern laws and intellectual property law. *This is precisely an ontogenetic developmental theory of evolution and knowledge generation*. I will describe the work of Hayek, in his most famous publication, *The Use of Knowledge in Society*,

3.2 The systemic and the actual problem

The common notion that the more relevant information we have means that we can evaluate risks and establish preferences which can then be used to determine means—*thus, the idea that any problem that we have is only a logical one*—is wrong (Hayek, 1945). Hayek (1945) said that this evaluation of best use of available means (resources) is part of our assumptions.

Hayek (1945) went on to say that the actual economic problem in society is that the current economic calculus is not suitable to solve this issue. This is because the “data” from which the economic calculus starts never reflects the whole society—*and could never be by a single mind at least*. Hayek (1945) notes how the issue with rational economic orders and structures is that the knowledge of relevant circumstances does not exist is concentrations or integrations, “but instead as dispersed bits

of incomplete and frequently contradictory knowledge which all separate individuals possess". Thus, the economic problem is not that of given resource allocation because given means a single mind which has tried to solve the problem created from the data he or she has used (incomplete information/knowledge). *The actual problem is how to secure the "best" uses of resources known to members of society in terms of their own relative ends which only they know*. Many of the economic problems in society, rest on the abovementioned mistaken economic assumption.

3.3 Planning and centralization

Hayek (1945) went on to examine the role of planning and centralization within economies. Planning refers to the complex and interrelated decisions regarding resource allocation within a society. The issue with planning (amongst other things) is that the central authorities *a priori* knowledge, which it uses to enactment mechanisms of planning, comes from someone else who must then try to convey this to the planner. The communication of this knowledge is a problem for which economic theories do not account. The best way to utilize knowledge initially dispersed among all people is the main issue of economic policy—and their designs of efficiency (Hayek, 1945). This, in turn, depends on *who is doing the planning*; not whether planning is necessary. The core question is w*hether planning should be done centrally by a sole authority* for the whole economic system, or *whether it should be divided amongst individuals.*

In modern contexts, planning is usually associated with centralization and sole authorities, with competition referring to decentralized planning by the many separate persons involved. In-between those is the monopoly—the delegation of planning to organized industries (Hayek, 1945). Determining which of these is most efficient and suitable depends on *which of them can be expected to use their existing knowledge more fully.*

This question depends on *whether we are more likely to give a central authority all requisite knowledge which should be used*, but which is initially spread or dispersed among many different actors or individuals. It also depends on *whether this authority can give those individuals additional knowledge which they will need* to allow them to adapt their planning in accordance with others (Hayek, 1945). Thus, the main idea is that *it is about information types, dissemination channels, and information asymmetries*—it is about *relativity*.

3.4 Relativity

The answer to the above depends on the different kinds of knowledge that one is dealing with. This also turns on the relative importance of different kinds of knowledge (those that we can expect to be in the possession of individuals and those which will more likely be in the possession of an authority made up of experts). It is an assumption today that the latter would be in a better position because of the influx and status that scientific knowledge has occupied (Hayek, 1945). However, scientific knowledge is neither the only form of knowledge, nor is it the most important. Scientific knowledge is not the collection of all knowledge, and it does not reflect all knowledge. For scientific knowledge, a centralized authority of experts is likely to be in the best position to command all the best knowledge available—but all this amounts to is a shift of the burden of the problem to experts.

3.5 Ontogenetic disorganized knowledge

Ontogenetic disorganized knowledge is not scientific (in that it is generalizable), but it is just as important precisely because of its particularity. It is the knowledge of particular times and places—

the knowledge of individualized contexts. This is the kind of knowledge in which individuals have advantages over others because they have unique, and possibly beneficial, information. *But it will only be beneficial if the decisions depending on it are left to the individual or are made with their active participation and co-operation* (Hayek, 1945). This kind of information has an equalizing effect. This is, in effect, practical knowledge or skills. It is that knowledge which is acquired on the job and is not of a theoretical basis—*it changes constantly*. It consists of knowledge of people, local conditions, special circumstances, and strategy (social, *sui generis* knowledge)—and it is valuable to those who create and use it. It also presents itself as the *knowledge of momentary opportunity and alternative techniques, which is unknown to others at a specific time* (Hayek, 1945). It is a knowledge of action! Unfortunately, it is the case that opportunistic is reflected in a negative light—*this kind of knowledge is bad*—but why? It is because of the idea that advantage to one must mean that that someone must be dishonest or have acted in a dishonorable way with regards to someone else—*he must have duped someone else to get an advance* (Hayek, 1945). Yet, this is precisely what must happen—society *must* make the best use of available knowledge and resources, just as society *must make use of the latest scientific theories*. This kind of thinking infiltrated commerce more so than it has production. Unfortunately, many think that knowledge must be "given" instead of *created*. This leads to thinking that all knowledge must be readily available to everyone, and arguments against existing governance are usually directed at knowledge *not being readily available to all*. However, this ignores everything that I have mentioned above.

This is part of a larger problem, and that is the smaller attribution to change. At policy level, there are only a few small points of difference between a central authority and its political or economic opposition—small alterations with no real substance as to production planning (Hayek, 1945). It is fundamentally not possible to plan far ahead, or even to plan just past one's nose. If a plan succeeds, it is more driven luck than anything else. The smaller attribution to change can be attributed to the creation of a new set of problems—*if we keep existing orders, then we can keep unsuccessfully fighting the same problems instead of creating new ones*. Thus, no need to expend energy and re-plan. Unfortunately, this resistance to change is present in economic thinking; and it is the same people who argue that economic knowledge has taken a backseat to technological knowledge that also argue against change. It is not true that with modern technological achievements economical decisions should only be required in long intervals because there are small changes occurring at all times on the ground.

One of the reasons why this happens is because of the obsession with statistical aggregates—we tend to ignore small changes. These aggregates show stability—the detail does not. These aggregates often rely on the "law of large numbers" or compensatory absorption of changes. However, this stability is a farce. *Aggregates tell us the lies that we would like to hear when we plan*. The continuous flow of goods and services, for example, is maintained by constant and deliberate adjustments; decisions made daily based on facts which change continuously (Hayek, 1945). *Opportunity and risk lives on the ground. This kind of changing knowledge is not able to be represented statistically because of its nature* (Hayek, 1945).

If this kind of knowledge cannot be represented statistically, then it also cannot be conveyed to a central planner or authority. One cannot represent rate of change in different variables and parameters such as date, time, place, context, and quality—*precisely because they are particulars*. Even if a central mind had all the information of small events or changes, it would not be able to adjust through re-evaluating all other interconnected effected knowledge and relations. To account for this, central planners use rates of equivalence consisting of values and marginal rates of substitution. They

attach numeral indexes to scarce resources that are not derived from any property possessed by that particular thing or resource. This index reflects a condensed form of its significance in terms of the whole system of relations, means, and ends (Hayek, 1945). *Thus, central planners must find a way in which to leave these kinds of planning decisions to those on the ground* (Hayek, 1945).

The main issue in society is that of rapid adaptation to changes on the ground. We must then leave the ultimate decisions to those on the ground who are familiar with these contexts and circumstances. This is because these folk know the circumstances, they know the relevant changes, the resources directly available to them, and how best to use them (Hayek, 1945). Society cannot wait for a central authority to have this knowledge communicated to them, and hen to issue directives—that will not work in terms of the framework above. Decentralization was the solution.

3.6 Decentralization of ontogenetic knowledge

The decentralization of ontogenetic knowledge allows for proper usage of this kind of knowledge, but this does not solve everything. The man on the ground needs communication of non-intimate information so that he may better make decisions in terms of the whole. This raises questions of: How much knowledge does he need? What knowledge does he need? When does he need it? Truth be told, everything has an effect making all events *prima facie* relevant. However, relevancy should be evaluated according to ease or difficulty—*how much easier will it be to get A, or how much more difficult will it be to get A? What is in demand right now? What is the suitability of the alternatives?* The man on the ground does not need to know the why's, just the relative direct effect on his product or needs in his environment (Hayek, 1945). In other words, *self-referential effects*.

3.7 The price system as the solution

The economic calculus involved in the price system solves these issues—the man on the ground can rely on the system of numerical indexes mentioned above. In the event of small changes within his environment, he only needs to consider these quantitative indexes which contain concentrated information, including that which is relevant to him. He can then adjust each of his dispositions accordingly without needing to know all the information that led up to this change (Hayek, 1945). Price systems can co-ordinate dispersed facts or knowledge by separate persons, in the same way as the values aid individuals in coordinating their plans (Hayek, 1945). One does not need to know the causes in order to enact appropriate responses by using this system. The enaction then has a reaction—it acts as a cause, which is then reflected in the system too, and an ongoing cycle ensues. The whole acts as a single market, not because members evaluate the whole body of information, but because their limitations are shared and overlap with each other—thus, they all receive the same information. There is informational symmetry with regards to communicating relevant information (Hayek, 1945). There is dialectic relationship between all intermediaries and the price system. The fact that there is a single value for an item, and this is connected to other things (such as transport costs), acts as a dynamic depressionary mechanism of relevant information for decision-making.

The most important aspect of this system is the economy of knowledge which it uses as its basis to supply only the relevant information to participants to make correct decisions for themselves. The values, or symbols, as abbreviated forms of information allow for only the most essential information to be communicated to relevant participants (Hayek, 1945). It induces and allows for the registering of ongoing changes. The price mechanism is thus not about providing perfect knowledge; *it is about demarcating rates of change from which relevant information can be abstracted. Its strength lies in*

its flux—the more rigid the price system becomes, the less useful it is. It produces a group of movement in directions. More so, its beauty lies in the fact that it is not of human design—*it evolved without human design* (Hayek, 1945). This lack of design of the price system tends to be a source of anxiety. Many may argue that it cannot be co-incidental that this system is central to modern society, but this is incorrect. It is actually the other way around; man discovered the system without understanding it, *and modern civilization was created because of it* (Hayek, 1945). We must remember the exact problems we needed to resolve: (1) manners in which to extend the range of use of resources beyond the control of a singular mind/entity; (2) avoiding conscious control by a singular mind/body; and (3) how to induce individuals to do desirable things without anyone needing to tell them what to do (Hayek, 1945). *We do not need to understand the price system to make use of it. It has enabled an adequate division of labor and a coordinated utilization of resources based on equally divided knowledge*. Individuals are free to decide on their own pursuits and apply their own knowledge, skills, and labor. As individuals freely pursue their own interests, they also do what is in the general interest (Hayek, 1945).

The price system reflects just one mechanism which is contra the usual position of "thinking about things" or "careful consideration". *Most of the jumps in society come from extending the number of important operations that we can perform without thinking about them* (Hayek, 1945). *This includes language and much of our cultural and social endowments*.

4 Part D: John Locke

In Locke's *Essay on Human Understanding* (1860), he described the *substantial* and the *essential* self, and distinguished between real and nominal essences. In his discussions on this, he alleged that real essences will be unknowable, as they are at the corpuscular level to which there is no access. Nominal essences on the other hand are those which arise through experience including language, cultures and the contents of consciousness which forms through nominal experiences and memory (Locke, 1860). In the Essay, he explicitly argues that the mind resembles a mirror, which simply fixes the object (reflects) of its reflection. In other words, the mind is passive, and a *tabula rasa* (Abrams, 1953). The Lockian mind functions through passive perception (Abrams, 1953), wherein it passively receives completed images from the external which then imprint sensations on it. Locke is also the progenitor of the concept of qualia, which arose as the indivisible remainder of the very structure of the mind he proposed. Without said passive structure, *qualia may not be said indivisible remainder*.

Importantly, the active interpretive, and not passive mind has been confirmed by neuroscience and neurobiology through the establishment of "interoception" (Sapolsky, 2017). Nonetheless, consciousness has been a hotly debated topic for some-time, which Naidoo (2023b) characterizes as being a state of affect. Affect, according to Spinoza, is emotion, which is (e)nergy-in-(motion). According to Hawkins and Dawkins (2021), consciousness arises when the brain fails to make a correct prediction Hawkins and Dawkins (2021) also note that the brain "votes" on the perception we experience. Hence, as Naidoo (2023b) has averted, consciousness is a heightened state of affect, or energy, which arises in a failure to predict, to supply the necessary energy for a correction of said failed prediction, which requires a world-map-update. This explains the process, purpose and what consciousness qualitatively is; it is a *limit*.

In keeping with Locke (1689), his *Two Treatises of Government* ushered in an important political shift. Locke (1690) said the following of governments:

> "Though the Earth, and all inferior Creatures be common to all Men, yet every Man has a Property in his own Person. This no Body has any Right to but himself. The Labour of his Body, and the Work of his Hands, we may say, are properly his. Whatsoever then he removes out of the State that Nature hath provided, and left it in, he hath mixed his Labour with, and joyned to it something that is his own, and thereby makes it his Property".

Lockian ideology reflected and precipitated a time of change. This ideology shaped the law, societal views and norms, and the bore the justification of property, as being one's labor. The influence of Locke's passive mind, and his private property ideology was immense (Rose, 1988). From this, bore the zeitgeist of "possessive individualism" (Macpherson, 2011).

Importantly, Locke's ideology shaped notions like creativity, labor, originality, and proprietorship. Locke's thesis of the sovereign was essentially a defense of private property in which he posits a natural right of men to property which is not dependent on the existence of any government. Despite Locke (1690) publishing his treatise after the inception of letters patent, it had a monumental impact on governance and the attitudes of society. Locke (1690) divorced property rights from the sovereign or governments, and also removed any natural law limitations on these property rights. As Rose (1988) notes, the cultural attitude of society reflected a different view of property. Society now felt inclined to their labors as being property. Thereafter, began a series of social and legal challenges that would forever shape intellectual property, and the world at large. This is reflected in the literary debates.

5 Part E: Freedom and objects

Kant's well known doxa of *Du kannst, weil Du darfst, was Du willst*, means that *you can because you must*. This is the essence of Kantian freedom; namely following a duty (in terms of the categorical imperative) even where one wishes not to. There are two interpretations of this, the first is the orthodox, or naïve way, where the Kantian *an sich* are treated in the literal. The second way, is far more insightful, and better integrated, given Kant's body of work (Naidoo, 2023a). All that is necessary to know here, is that Kant was trying to safeguard freedom, by placing the subject *within a constitutive gap*.

Whilst Kant is often interpreted as contra freedom-and pro duty; this was a misreading, I suggest. In *The Abyss of Freedom*, Žižek (1997) describes Kant as being the foundational thinker of autonomy and freedom, since it was Kant who put the first crack in the concept of the universal. In *The Puppet and the Dwarf: The Perverse Core of Christianity*, Žižek (2003) outlines the reasons for this. The noumenal world, the *ding an sich*, is posited as pure freedom. However pure freedom amounts to no freedom at all. Kant states that if noumenal subjectivity were to be accessed by reflective self-consciousness, this would be an awful majesty (*furchtbaren majestat)*. For Kant, pure freedom describes the subject as being a puppet on a string, having no agency. So, to counter this, Kant had to ensure that the subject was disconnected from the *noumea*, hence providing a grounding for freedom and autonomy. One is only free, insofar, as one is disconnected, or ignorant of, pure freedom, or the Divine Will. Phenomenal reality thus must be barred (incomplete or containing an irreducible link or deadlock). In other words, there must be a gap, or separation. Kant was trying to safeguard freedom, through maintaining a distance between the ultimate freedom, which amounts to no freedom. Kant was trying to guard against what we experience in society today; *freedom without the gap.*

In the *Critique of Pure Reason*, Kant (1890) created a form of judgement called the *infinite judgement*, which is not the affirmation or negation of a predicate, but rather the affirmation of a non-predicate. An infinite judgment is one in which a qualitative finding of "existence" cannot be attached as a predicate. This is perfectly analogous to Gödel's incompleteness theorem (Naidoo, 2023b). Kant's (1890) aim was to safeguard said constitutive gap, without which, produces the kind of freedom we find in liberalism today. That is, *freedom-without-the-gap*, which is an *illusion of freedom*. In liberalism, freedom is espoused as being enhanced and protected by *increased participation*, in the sense that there is increased choice to make amongst objects or creations, thus allowing one to pursue one's own ends in life, and obtain what one wishes. Freedom is thus *attached to objects*. However, while the magnitude of choices may seem to be increasing, the actual concept of "freedom of choice" is being degraded. In *Das Kapital,* Karl Marx (1887) called this *ideological reification,* which is the necessary condition of outsourcing one's own exploitation, which is then rebranded psychologically as freedom, thus hiding the fact that *there was no real choice to begin with*. Žižek (1989) says the following:

> "This procedure thus implies a certain logic of exception: every ideological Universal - for example freedom, equality - is 'false' in so far as it necessarily includes a specific case which breaks its unity, lays open its falsity. Freedom, for example: a universal notion comprising a number of species (freedom of speech and press, freedom of consciousness, freedom of commerce, political freedom, and so on) but also, by means of a structural necessity, a specific freedom (that of the worker to sell freely his own labour on the market) which subverts this universal notion. That is to say, this freedom is the very opposite of effective freedom: by selling his labour 'freely', the worker loses his freedom - the real content of this free act of sale is the worker's enslavement to capital. The crucial point is, of course, that it is precisely this paradoxical freedom, the form of its opposite, which closes the circle of bourgeois freedoms."

In other words, *people are free to sell their labors, but not the products of their labor itself.* Labor has in this sense, become a commodity, and the equivalent exchange becomes its own negation; the form of exploitation is the appropriation of *surplus value* (Žižek, 1989). As Žižek (1989) rightly points out, this *negation is internal to equivalent exchange, and not its violation*. Exploitation is not a lack adequate recompense (the exchange between labor and capital is equivalent and equitable). It is the fact that labor becomes a commodity which produces a surplus value, *and this surplus value is what is appropriated within capitalism*.

Surplus value (or *jouissance*, in Lancanian parlance (Naidoo, 2023b)) is abstract. It is the gain of something else; this something else is the gain of *psychoanalytic enjoyment.* This enjoyment pertains to a knowledge asymmetry, in one's favor, instead of the other person or people.

In Hegelian dixit (2018), it is the knowledge of being the Master. Lacan (2006) elaborates this further in his allegory of what it means to be a King. Lacan (2006) says that *a King who thinks he is the King is the true fool*; he can never prove that he is King, because if he does prove it, he ceases to be the King. The King, as power or independent identity, is nothing more than a fetish—*a surplus enjoyment*. Ultimately, there is a misrecognition on the part of the King's subjects. *They think that they are subjects of the King, who is King simply because he is King. In actuality, the King is King because of this misrecognition by the people; without which there is no King*. It is not the subjects who are the subjects of a King; *it is the King, who is a subject of the subject's misrecognition.* The surplus enjoyment lies with the King, since there is an asymmetry of knowledge in his favor. In other words, *the King obtains enjoyment, by knowing what his subjects think they (the subjects) know.*

Going back to the commodity trade fetish – this is not a truly free society. As Althusser (2014) pointed out, freedom is chained to "free actions" amongst people in a marketplace of commodities or objects. We are free in so far as we are able to contract with others; to get what we want from them. Their value, and ours is tied to what they have. What others have, structures our desires. *It is a marketplace structured on differences in commodities*. Freedom then is intricately linked with objects; not opportunity or choices in a substantial sense, but rather a choice on what there is on offer today in the cafeteria of the market. The fetish then lays not with an inter-subjective freedom (relations between people); but rather relations between things *which are disguised as relations between subjects* (Žižek, 1989). Of course, what there is on offer today is then linked to the Master; and the laws structured around the markets.

This links to the liberal notion of "bootstrapping", or the notion of the "democratic-self-made-man." Namely, one can be or do anything one wants, as long as one works hard enough. This is analogous to the marketplace of ideas, where people bootstrap, and compete with one another, to obtain a particular status within said market, or society. This is also why, politics, science, social concerns, and philosophy cannot be de-linked. The scientific method is liberalism, but in terms of knowledge, instead of human bodies.

6 Part F: The common law and intellectual property

6.1 Millar v Taylor

The judgement dealt with the claim to a perpetual copyright (at the time, the copyright was a common law right), in sentiment/style/ideas. This is the first case, in a series of important cases, wherein intellectual property law was fished out, to be what it is today – namely, a limit to the common law of property, which serves to maintain a legal, and societal dynamic equilibrium. Below I present some important snippets from the case, which are entirely transposable to the debate today regarding the governance of information and data. *Intellectual property was formed as the solution to these issues*.

6.1.1 The UK common law (JA)

The English common law is founded on the laws of nature and reason (natural law). The basis of the common law comes from different sources, including natural and moral philosophy, civil and cannon law, logic, the uses associated with customs and conversations between the common people and their opinions, and the general nature of humankind. One of the maxims is *quod tibi fieri non vis, alteri ne feceris* (do unto others as you wish done to you). Before Edward the Confessor, the common law was called the "folcright" or the peoples right or common justice/right. In other words, the common law represents the universal interest to serve the interest of mankind itself.

6.1.2 The nature of the common law of property (Justice Yates)

For something to be common law property, it must be a chattel. Chattels consists of goods, debts, and contracts (Millar v Taylor, 1769). The claim herein is neither a debt nor a contract, therefore it can only be a chattel. This means that it must fall under goods. Goods however *must be capable of possession and have visible substance—as only things of visible substance are capable of possession*. An unpublished manuscript will qualify as a good because it is corporeal; however, after publication *the ideas are incorporeal and therefore incapable of distinct and separate possession* (Millar v Taylor, 1769). Ideas cannot be seized, forfeited, or possessed. If they are to be property, they *must be*

subject to the general laws of property which apply to all persons. These are foundational to law itself. The sole right of publishing cannot be confined to the author (of those ideas). The plaintiffs claimed that this is a special right which speaks to a particular interest or a privilege. However, Justice Yates (JY) noted that only an inheritance satisfies this. All personal property is thus total and absolute (Millar v Taylor, 1769).

JY goes on to say that the right claimed cannot even have an existence in the common law of England—as the whole right claimed for *is a right of action*; a right of bringing an action against those that print the author's work without his consent (Millar v Taylor, 1769). This is vindicative; it *is an action in personam and not an action in rem*. The maxim of the law which says that things in actions are not assignable (Millar v Taylor, 1769). This action itself is founded on the right to sue. *As the law prioritizes peace, it is not assignable*. While the statutes may have made a new law and enabled assignment, this does not extend to the common law. Not even debts are assignable at common law (Millar v Taylor, 1769). The claim is a tort; torts are not assignable in law or equity. Therefore, it cannot be a common law right. It does not fall within any species of common law property either—therefore is not a common law right. The whole claim an author can make is by public benevolence, in order to encourage him; but it is not an absolute or a coercive right (Millar v Taylor, 1769). The author's right is exactly that of the inventor. Every purchaser has the right to make use of it as he pleases.

Improvement in learning was not part of the thoughts or attention of our ancestors. The invention of an author is thus a species of property unknown to the common law of England (Millar v Taylor, 1769). Its uses are immemorial, and the views of it tend to benefit and advantage the public with respect to the necessaries of life, and not to the improvement and graces of the mind. Hence it is not part of the common law of England.

When the nation was liberalized and became established and organized, it became the responsibility of the legislature to make provisions of this nature for encouragements they saw fit (Millar v Taylor, 1769). They have done so by the Statute of Anne. The Act should be the sole point of reference, not these other authorities to which the plaintiff referred. This Act was made by a legal and regular authority without the mixture of political views (Millar v Taylor, 1769). The plaintiffs knew that this statute was decisive against them—that is why they avoided it. The design of the Act was to vest a temporary copyright in authors, and to establish that right for a limited time. This common law right claimed would make the Act a nullity (Millar v Taylor, 1769). The title of the Act seems to JY to be intentionally designed to vest property only for a limited time. The title clearly implies that there was no right prior to it. Thus, they intended to give them some security which they did not have before. If the common law right did exist, people would just waive the benefit of the Act—which was worried about monopolies and exorbitant prices (Millar v Taylor, 1769). Thus, for these reasons it is not part of the common law.

6.1.2.1 Conflation of personal rights with objects of property

JY rejected the argument put forward by the plaintiffs; namely that the authors had a "*jus utendi et fruendi*" claim in the work. This was a conflation between rights in productions of labor and the concept of personal dominion. Being within the personal dominion is neither a determinant, nor is it the same as an object of property (Millar v Taylor, 1769). *The maxim so claimed is to protect the proprietary space of subjects, and not to be used to determine an object* (it is the extent of a subject to be a proprietor).

6.1.3 The foundations and general rules of property law (JY)

JY points out that *exclusiveness* is fundamental as a foundational element which fulfils the private element inherent in the concept of private property. *Dominion does not simply speak to within the control of the author—it also inversely means that it must not be within the control of someone else*. The plaintiff countered this, saying that exclusiveness was evident by the right of first publication which vested in authors. However, JY said that while this may be so, it is only after the fact of printing (Millar v Taylor, 1769). Importantly for JY, the essentials (or *essentialia*), or the substance of a thing must, in its whole, belong to one person—*to the exclusion of others*. This must also apply to ideas and sentiments—for the sake of the rule of law. How can ideas and sentiments be privatized?

JY then runs through the general legal rules for private property. First, if there is no property, there is no injury. All objects of property must be corporeal—and this is the necessary nature of property. This is because the object must be *visible for property to have a distinct and separate possession*. It must also have bounds—being a beginning and an end—and it must be distinguishable by some mark (Millar v Taylor, 1769).

> "It must be something that is visibly and distinctly enjoyed; that which is capable of all the rights and accidents and qualities incident to property: and this requires a substance to sustain them" (Millar v Taylor, 1769).

The claim here however is to style and ideas (Millar v Taylor, 1769). These are ideals which have no bounds or distinguishability. They cannot be visibly possessed, and they do not have any substance which can satisfy the necessities or qualities of legal private property. They exist purely in the mind, and they are incapable of other modes of acquisition or enjoyment other than a conceptual mental possession or apprehension. Surely then argues JY they cannot be infringed upon; after all, how can one trespass against something of this nature?

He then goes on to distinguish between incorporeal property and incorporeal rights. The latter is allowable as property, but the former is not. Furthermore, all incorporeal rights are attached to something corporeal as far as he is aware.

> "No right can exist, without a substance to retain it, and to which it is confined: it would otherwise, be a right without any existence" (Millar v Taylor, 1769).

JY goes on to say that *profits of publication are not objects of property either because they are uncertain, incidental, and causal (to publication)*. The profits would depend on the object and the object is defined by its composition. Thus, authors must prove that they have a common law perpetual right to the object of the property *and then only can he claim a right to its profits*. To do this, *it must first be proved that there is a property interest*. This interest must be in excluding others from it—without that, any claimed property right is not a right to legal private property. In support, he quotes another maxim—"nothing can be an object of property, which is not capable of a sole and exclusive enjoyment" (Millar v Taylor, 1769). To summarize, one cannot claim something as theirs if they cannot prevent others from using it.

6.1.3.1 Effect of publication on property (JY)

JY reasons that when ideas are published, they are no longer within one's possession. He uses the quote from the *Institutes* relating to wild animals, which stipulates that if an object is not in your possession, then it is no longer yours. Thus, for JY, when ideas are published, they are no longer

capable of being objects of property, and mankind is entitled to read them (Millar v Taylor, 1769). The act of publication by an author is a gift to the public. The plaintiffs argued against this by saying that an author's publication is not to be construed as giving them the property—but to peruse it. They used the example of giving the public tickets to the opera. The ticket allows access, but not the ability to print other tickets (Millar v Taylor, 1769). JY rejected this because this is not applicable to the context—being general publication. It is thus open to mankind. When an author empowers a bookseller to sell the book, inherent is conveyance of the generality of the property (Millar v Taylor, 1769). Authors cannot alter the fixed constitution of things and a man cannot retain what he has parted with (Millar v Taylor, 1769).

6.1.3.2 Rejection of value (JY)

JY argued that it is difficult to ascribe value to incorporeals if they are detached from the manuscript and post publication. He says that the relied upon right of sole publication is *based on the value to the author*. This value itself is not indicative of property in incorporeals. He gives the example of sunlight and air, both of which have value but are not private property (Millar v Taylor, 1769). Instead of value, he contends that exclusiveness is the actual foundation (as above).

6.1.4 The purpose and legal consideration of property law (JY)

The common justification is that private property arose to preserve peace, "which could not exist in a promiscuous scramble". From this purpose arose a moral obligation binding all to not intrude on the possessions of others (Millar v Taylor, 1769). *This obligation extends (and is only possible) where objects are distinguishable to all men (indica), so that people can act with intentionality as required by the law*. He then quotes another maxim of property which says that nothing can be an object of *property if it is incapable of distinguishable proprietary marks*. It is thus a legal requirement that people must know their duties in order not to breach them (Millar v Taylor, 1769). *Indica* is necessary. The law works on the basis of will/intents of another; the law cannot hold someone guilty of a crime if he did not know that the object belonged to another.

Responding to claims that *indica* could be satisfied by the inclusion of authors names on the title page, JY said that this was not sufficient. This is because many of the best authors published their works, not for pecuniary profit, but for fame and honor. Others have also written for the benefit of mankind. Another legal issue is the relinquish-ability or alienation of this claimed property. JY argued that it is impossible for them to be visibly relinquished *because there is no visible possession*. If there is no token of possession, then there is no distinguishability and thus no relinquishing. These rules are applicable to all other property—*therefore the rule of law requires that they apply to all*.

6.1.4.1 Rejection of ideas (JY)

The plaintiffs argued that an author has perpetual property in his style and to the ideas within the work. To this, JY stated that ideas are free (Millar v Taylor, 1769). Every man had this right to keep his own sentiments. They also had a right to determine whether to make them public or not. At the manuscript stage, this applies too. Only he can use it in the ways he wishes, including the right of first publication. If someone takes these rights away from him, they committed a legal wrong and the court can intervene (Millar v Taylor, 1769). However, a legal wrong has not occurred here because it was he who published and profited from it.

Further, the plaintiffs argued that both the invention and the labor of sole pains (Millar v Taylor, 1769) by authors in composition were the modes of acquisition to this perpetual right which is

claimed. JY agreed with this, but only insofar as the physical manuscript. This does not extend to ideas. Neither invention nor labor in the form of occupancy can be used to change the nature of things—nor to extend rights where the claimed property is *contra* the fundamentals and bounds of the scope of property (Millar v Taylor, 1769). Occupancy means to take possession (or discovering and defining vacant property). Labor, on the other hand, means the actual taking of possession of that property (Millar v Taylor, 1769). How can one take possession or occupancy in ideas of the mind? Appropriation is fundamental to remove things from the commons and into the exclusive domain or dominium of the proprietor. *This is of importance to the legal notion of private property* because occupancy supports the practical reason aspect of the law. Practical reason speaks to adequate communication and notification to others in a social environment. The communication must tell someone else that something is in the private domain of someone else. Thus, they must trespass against it. This outward mark of occupancy communicates the privatization of the thing. You cannot display this outward necessary mark of privatization to others with the so claimed (ideas and so forth) herein. Thus, it cannot be legal property (Millar v Taylor, 1769).

Also, JY raises the question of when would property rights attach or become active in ideas? For corporeals, this occurs with possession. But for authors, does this then mean that it activates when he ideates, or when he completes the idea, or when he writes it down in a private capacity? What would happen if another man had the same idea? How must that conflict be dealt with? Would someone else be precluded from publishing his same idea? How must the law deal with this conflict? Would someone else be precluded from publication of his idea because it existed in and was occupied by/in the mind of another (Millar v Taylor, 1769)? This situation and uncertainty is legally undesirable, and impossible for the law to contend with. It is completely impractical. The law prioritizes peace, certainty, possibility, and adequate communication; otherwise, it is not law.

6.2 Mind and Body: of Patents and Copyright

6.2.1 Biology versus mechanism

This distinction between mind and body can be traced back to the Aristotelian concept of form and unity within the biological paradigm. Biological material Aristotle argued (much like Kant), were both means and ends in themselves (in other words, reciprocally constitutive (see Naidoo, 2023d). Mechanical substances, which are covered by patents, is different, because mechanical structures are defined by the fact that the *parts come before the whole*. This notion also created a divide in the concept of labor. There was now mental labor and manual labor. Edward Young (1759), in *Conjectures on Original Composition*, differentiated between patent inventions and copyright works by analogy to a garden:

> "An Original may be said to be of a vegetable nature; it rises spontaneously from the vital root of Genius; it grows, it is not made: Imitations are often a sort of Manufacture wrought up by those Mechanics, Art, and Labour, out of pre-existent materials not their own" (Young, 1759).

6.2.2 William Warburton

William Warburton also had a massive impact. Warburton (1747) sought to protect his legacy from Alexander Pope. To do so, he crafted the following argument in *A Letter from an Author to a Member of Parliament Concerning Literary Property* (Warburton, 1747). Warburton (1747) argued that moveable property was divided into two categories of things (Rose, 1988): (1) natural; and (2) artificial. Artificial things were further divided into productions of mental activities (the mind) and

productions of manual activities (the body). The latter resulted in the creation of corporeal items like utensils (knives). The former, on the other hand, produced property in the form of literary works (manuscripts and the like); but it also produced property in the "*doctrine itself rather than the ink and paper on which the doctrine was inscribed*" (Rose, 1988). *This property was not limited to the single object unlike that of manual activities*. What Warburton (1747) is referring to is "likeness" or "identity".

Warburton (1747) conceded that mechanical inventions did have characteristics of both—thus, when a mechanical invention took the form of a utensil, the property interest should be that of *sole production* (Rose, 1988). To Warburton (1747), patents as special limited rights could be justified because of the marriage of the mind and manual labor. *Literary works, on the other hand, being purely of the mind, gave rise to a different and distinct interest* (Rose, 1988). Rose (1988) notes how Warburton's (1747) argument would not have envisaged the modern assembly line (or the industrial revolution that followed). Adam Smith (1776) later followed suit in his seminal work *The Wealth of Nations*, where he drew distinctions between mental (skilled) and manual (common) labor based on the "investments" one made in their education. For Smith (1776), this would draw the economic line (which spurred the later economic discussions of intellectual property, such as the rational of recouping financial investments and research and development).

Hence, given the cases, the law distinguished between mental and physical labor; privileging mental labor (Sherman and Bently, 2003). This shaped the discourse and the uniting value of various laws which governed intangibles (such as the Engravers Act of 1735) (Sherman and Bently, 2003). Regardless, it was in this debate that the substantive differences between authors and inventors, copyright and patents, the mind and the body took place. Proponents of the perpetual copyright used this diversion as a means in which to further their arguments (Rose, 1988).

6.3 Style as property?

Given the zeitgeist of pragmatism which enveloped society, elements of an author's *identity* became intertwined with the works produced. Style reflected the man, who produced the work. Style was not a reflection of the content per se, but rather the form that the content took. The language, grammar, punctuation marks, and use of creative tools in language (such as metaphors and similes) were all reflections of the man and his state of mind, rather than the subject matter of the work itself. Thus, individuality arose, and it became stronger in the 18th century.

What does style look like? Some may think style must be validated by external evidence, and, if it is not, then it is mostly conjecture. There are clues to identify style however, which take the form of the creative language tools mentioned above. These instances aid in identifying the man, since they reflect his tastes, feelings, and understandings of the world. How similes are treated, for example, demonstrates how they understand the objects of the simile. *We know authors through their styles. The style is the source of originality*. Warburton (1747) rejected Alexander Young's thesis; instead, he said that *it is the manner that gave rise to originality, not the matter*. Poetry is thus imitation, and the individual finds himself in the style. *Matter mirrors the world, and manner mirrors the man.*

This period also bore the seeds of objectivism, namely that there was a Universal good to art; a Universal perfection or beauty. Within pragmatism, this was the universalized and relatable human element contained in any work. The manner of communicating this universal element is subject to the variety of ways in which different authors can present it (genres, style and observations).

6.3.1 Tonson v Collins

The case of *Tonson v Collins* (1761) came before the Court of Kings Bench—in which Lord Mansfield sat. The opponents to perpetual common law copyright spoke of this property as being "ideal"—meaning not an exact copy of things in reality. Blackstone, acting for those pro-perpetual common law copyright, seemed to agree with this characterization when he said that "*one essential characteristic requisite of every subject of property was that it must be a thing of value*". He also said, "*that the value of a literary property lay wholly in the sentiment*" (Rose, 1994; Tonson v Collins, 1761).

Justice Yates, who played a key role in the development of copyright questioned Blackstone (1916) as to why there was a difference between copyright and patents. Justice Yates went so far as asserting that there could not be and should not be! Pushed into a corner, Blackstone (1916) invoked Warburton (1747).

Blackstone responded that there is a difference between mechanical inventions and literary works—he gave the example of two engines. While these two engines may resemble each other, they are not and could never be so, *because the materials and workmanship differ*. However, every duplicate of a literary text was the same text *because its essence was immaterial*. Warburton (1747), of course argued in his letters, that it was essence which characterized the essence of his books; this was a "doctrine"—a term equivalent to Blackstone's own earlier "sentiment".

As Dreazly (2003) rightly points out, Blackstone (1916) subtly shifted the characterization of the essence of a book from an "idea" to that of the fusion of idea and language. He says:

> "Style and sentiment are the essentials of a literary composition. These alone constitute its identity. The paper and print are merely accidents, which serve as vehicles to convey that style and sentiment to a distance. Every duplicate therefore of a work, whether ten or ten thousand, if it conveys the same style and sentiment, is the same identical work, which was produced by the author's invention and labour" (Tonson v Collins, 1761).

During the case, it became clear that there was collusion between both parties, and thus the court refused to give judgement. Nonetheless, this did not stop Blackstone (1916) from refining and codifying his arguments in his *Commentaries*, which became the leading legal work in England after its publication. In the second edition, Blackstone (1916) stated:

> "When a man by the exertion of his rational powers has produced an original work, he has clearly a right to dispose of that identical work as he pleases, and any attempt to take it from him, or vary the disposition he has made of it, is an invasion of his right of property. Now the identity of a literary composition consists intirely in the sentiment and the language; the same conceptions, cloathed in the same words, must necessarily be the same composition: and whatever method be taken of conveying that composition to the ear or the eye of another, by recital, by writing, or by printing, in any number of copies or at any period of time, it is always the identical work of the author which is so conveyed; and no other man can have a right to convey or transfer it without his consent, either tacitly or expressly given".

One can see how his phrasing gives a distinct feel of corporeality to the work claimed. Blackstone (1916) said that the identity of literary property lay in its sentiment and language. Literary property was thus seen as a landed estate (Rose, 1994).

7 Part G: A map for readers

This manuscript began with uncovering the some of the hidden links structing all knowledge and society today. These hidden links are the philosophical underpinnings of Cartesian and Kantian epistemology. The importance of this discussion is the movement that I wished to facilitate from disembodied logic (Kant and Descartes), to embodied logic (Hegelian). Embodied logic is what will be used to create the framework for the information ontology. Moreso, embodied logic is what ensures that the concept of “open” is maintained and protected, which is central to liberalism and liberal democracies. This discussion is lead under the theme of the knowledge society. The notion of openness once more results in healthy and liberal society, and openness requires the free flow of knowledge in society.

Thereafter, I provided evidence for incorporating human experience and emotion into all knowledge bases (and thus disciplines), by highlighting the fundamental role that uncertainty and surprise plays (with reference to the information sciences). This supports my logic of embodiment, given that embodiment requires human experience and emotion. In highlighting the fundamental role of human subjectivity, I also validated Freudian thought on the importance of subjective activity (or active mind), in constructing knowledge, thus demonstrating that human experience and emotion can never be removed from any form of knowledge or discipline.

Using the ideas, understanding and arguments presented above, I then began the construction of an information ontology. I then applied other important concepts, such as embodiment to instances of knowledge organization within society, and how embodiment functions in how society views and treats objects. This is relevant for the discussions I lead on the imagination and the concept of openness once more. To ensure this kind of openness, I demonstrated how knowledge and truth are organized in society. These discussions also referenced Kant for example, who put a crack in the notion of purity/universals/objectivity/subjectivity.

I then continued to build the framework of the information ontology, based on more technical aspects of information, drawn from various sources. This all culminated with the information ontological framework, which was then used to answer questions relating to data governance.

8 References

Abrams, M. H. (1953). *The Mirror and the Lamp: Romantic Theory and the Critical Tradition*. Oxford: Oxford University Press.

Althusser, L. (2014). *On the Reproduction of Capitalism: Ideology and Ideological State Apparatuses*. London: Verso.

Bennett, C. H. (1973). Logical reversibility of computation. *IBM Journal of Research and* Development, 17 (6), 525–532. Doi: 10.1147/rd.176.0525

Blackstone, W. (1916). *Commentaries on the Laws of England (1765-1769)*. San Francisco: Bancroft-Whitney Company.

Bouwsma, W. J. (2020). Calvinism. *Britannica.* https://www.britannica.com/topic/Calvinism#:~:text=Calvinism%20%2C%20the%20theology%20advanced%20by,characteristic%20of%20the%20Reformed%20churches [Accessed March 17, 2023].

Byjus. (n.d.). Bayes' theorem. https://byjus.com/maths/bayes-theorem/ [Accessed August 5, 2023].

Deazley, R. (2003). The myth of copyright at common law. *Cambridge Law Journal*, 62(Part 1), 106-133.

DeWeese, M. R., and Meister, M. (1999). How to measure the information gained from one symbol. *Network*. 10 (4), 325–340.

Engraving Copyright Act 1734 (8 Geo. 2 c. 13).

Hawkins, J., and Dawkins, R. (2021). *A Thousand Brains: A New Theory of Intelligence*. New York: Basic Books.

Hayek, F. A. (1945). The use of knowledge in society. *American Economic Rev.* 35 (4), 519–530.

Hegel, G. W. F. (2018). *Phenomenology of Spirit* (translated by T. Pinkard). Oxford: Oxford University Press.

Hossain, I., and Blanchard, G. J. (2023). Ionic liquids exhibit the piezoelectric effect. *Journal of Physical Chemistry Letters* 14 (11), 2731–275. doi: 10.1021/acs.jpclett.3c00329

Hossenfelder, S. (2016). The remote Maxwell demon as energy down-converter. *Found. Phys.* 46, 505–516. doi: 10.1007/s10701-015-9981-7

Hossenfelder, S. (2014). The remote Maxwell Demon. *Back ReAction*. http://backreaction.blogspot.com/2014/12/the-remote-maxwell-demon.html [Accessed December 16, 2022].

Hossenfelder, S. (2018). *Lost in Math: How Beauty Leads Physics Astray*. New York: Basic Books.

Jameson, F. (1973). The vanishing mediator: Narrative structure in Max Weber. *New German Critique* 1, 52–89. doi: 10.2307/487630

Kant, I. (1890). *Critique of Pure Reason* (translated by J. M. D. Meikelejohn).

Lacan, J. (2006). *Écrits: The First Complete Edition in English* (translated by B. Fink). New York: W.W. Norton and Company.

Landauer, R. (1961). Irreversibility and heat generation in the computing process. *IBM Journal of Research and Development*, 5 (3), 183–191. Doi: 10.1147/rd.53.0183

Levi-Strauss, C. (2011). *Tristes Tropiques* (translated by J. Weightman and D. Weightman). London: Penguin Books.

Locke, J. (1860). An Essay Concerning Human Understanding. http://www.philotextes.info/spip/IMG/pdf/essay_concerning_human_understanding.pdf [Accessed January 18, 2023].

Locke, J. (1689). *Two Treatises of Government*.

Locke, J. (1690). *Second Treatise on Civil Government*. London: George Routledge and Sons.

Macpherson, C. B. (2011). *The Political Theory of Possessive Individualism: Hobbes to Locke*. Oxford: Oxford University Press.

Marx, K. (1887). *Capital: A Critique of Political Economy* (translated by S. Moore and E. Aveling). https://www.marxists.org/archive/marx/works/download/pdf/Capital-Volume-I.pdf

Millar v Taylor (1769) 4 BURR 2303.

Moreno, A., and Mossio, M. (2015). *Biological Autonomy: A Philosophical and Theoretical Enquiry*. Dordrecht: Springer.

Mukherjee, S., Asnani, H., and Kannan, S. (2019). CCMI: Classifier based conditional mutual information estimation. doi: 10.48550/arXiv.1906.01824

Naidoo, M. (2023a). An (ontogenetic)transcendental (de)materialist theory of subjectivity. doi: 10.31219/osf.io/cwugk
Naidoo, M. (2023b). Contradiction and desire. Doi: 10.31219/osf.io/up5f4
Naidoo, M. (2023c). The hard problem, qualia, agency, intelligence, and Freud. doi: 10.31219/osf.io/gqspk
Naidoo, M. (2023d). What does it mean to be an Agent? doi: 10.31219/osf.io/evna6. *Submitted to Frontiers.*
Popper, K. (1945). *The Open Society and Its Enemies*. London: Routledge.
Prior, A., and Geffet, M. (2003). “Mutual information and semantic similarity as predictors of word association strength: Modulation by association type and semantic relation,” in *Proceedings of Eurocogsci 03*, eds. F. Schmalhofer, R. M. Young, G. Katz, K. Graham (New York: Routledge).
Rose, M. (1988). The author as proprietor: Donaldson v. Becket and the genealogy of modern authorship. *Representations*, 23, 51–85. doi: 10.2307/2928566.
Rose, M. (1994). *Authors and Owners: The Invention of Copyright*. Cambridge: Harvard University Press.
Rowlands, P. (2007). *Zero to infinity: The foundations of physics*. World Scientific Publishing Company.
Salazar, D. (2022). Correlation is not correlation. https://david-salazar.github.io/posts/fat-vs-thin-tails/2020-05-22-correlation-is-not-correlation.html [Accessed April 28, 2023].
Sapolsky, R. M. (2017). *Behave: The Biology of Humans at Our Best and Worst*. New York: Penguin Books.
Shannon, C. E. (1940). *A symbolic analysis of relay and switching circuits* (PhD Thesis, Massachusetts Institute of Technology).
Shannon, C. E., and Weaver, W. (1964). *The Mathematical Theory of Communication*. Illinois: The University of Illinois Press.
Sherman, B., and Bently, L. (2003). *The Making of Modern Intellectual Property Law: The British Experience, 1760–1911*. Cambridge: Cambridge University Press.
Smith, A. (1776). *The Wealth of Nations*. New York: P. F. Collier & Son.
Taleb, N. N. (2007). *The Black Swan: The Impact of the Highly Improbable*. New York: Penguin Books.
Tonson v Collins (1761) ER 96.
Uebel, T. (2020). Vienna circle. *Stanford Encyclopedia of Philosophy*. https://plato.stanford.edu/entries/vienna-circle/ [Accessed February 10, 2023].
Warburton, W. (1747). *A Letter from an Author to a Member of Parliament Concerning Literary Property*. London.
Weisstein, E. W. (2023). Covariance. *MathWorld*. https://mathworld.wolfram.com/Covariance.html [Accessed April 21, 2023].
Wiener, N. (1961). *Cybernetics: Or the Control and Communication in the Animal and the Machine*. 2 ed. Cambridge: MIT Press.
Young, E. (1759). *Conjectures on Original Composition*. Manchester: University Press (1918).
Žižek, S. (1989). *The Sublime Object of Ideology*. London: Verso.
Žižek, S. (1997). *The Abyss of Freedom*. Michigan: University of Michigan Press.
Žižek, S. (2003). *The Puppet and the Dwarf: The Perverse Core of Christianity*. Cambridge: MIT Press.
Žižek, S. (2008). *The Ticklish Subject: The Absent Centre of Political Ontology*. London: Verso.
Zucker, S., and Harris, B. (n.d.). The Protestant Reformation. *Khan Academy*. https://www.khanacademy.org/humanities/renaissance-reformation/reformation-

counterreformation/beginner-guide-reforrmation/a/the-protestant-reformation [Accessed May 8, 2020].
